# Myeloperoxidase-anchored ENO1 mediates neutrophil extracellular trap DNA to enhance Treg differentiation via IFITM2 during sepsis

**DOI:** 10.1172/JCI183541

**Published:** 2025-09-02

**Authors:** Yi Jiang, Shenjia Gao, Xiya Li, Hao Sun, Xinyi Wu, Jiahui Gu, Zhaoyuan Chen, Han Wu, Xiaoqiang Zhao, Tongtong Zhang, Ronen Ben-Ami, Yuan Le, Timothy R. Billiar, Changhong Miao, Jie Zhang, Jun Wang, Wankun Chen

**Affiliations:** 1 Department of Anesthesiology, Zhongshan Hospital, Fudan University, Shanghai, China.; 2 Shanghai Key Laboratory of Perioperative Stress and Protection, Shanghai, China.; 3 Department of Integrative Medicine and Neurobiology, School of Basic Medical Science, Institutes of Integrative Medicine, State Key Laboratory of Medical Neurobiology and Ministry of Education Frontiers Center for Brain Science, Institutes of Brain Science, Shanghai Medical College, Fudan University, Shanghai, China.; 4 Infectious Diseases Unit, Tel Aviv Sourasky Medical Center, Faculty of Medicine, and; 5 Gray Faculty of Medical and Health Sciences, Tel Aviv University, Tel Aviv, Israel.; 6 Department of Anesthesiology and Hunan Province Key Laboratory of Brain Homeostasis, Third Xiangya Hospital, Central South University, Changsha, Hunan, China.; 7 Department of Surgery, University of Pittsburgh, Pittsburgh, Pennsylvania, USA.; 8 Department of Anesthesiology, Shanghai Geriatric Medical Center, Shanghai, China.; 9 Department of Anesthesiology, Qingpu Branch of Zhongshan Hospital Affiliated to Fudan University, Shanghai, China.

**Keywords:** Immunology, Infectious disease, Immunotherapy, T cell development

## Abstract

Sepsis is a life-threatening disease caused by a dysfunctional host response to infection. During sepsis, inflammation-related immunosuppression is the critical factor causing secondary infection and multiple organ dysfunction syndrome. The regulatory mechanisms underlying Treg differentiation and function, which significantly contribute to septic immunosuppression, require further clarification. In this study, we found that neutrophil extracellular traps (NETs) participated in the development of sepsis-induced immunosuppression by enhancing Treg differentiation and function via direct interaction with CD4^+^ T cells. Briefly, NETs anchored enolase 1 (ENO1) on the membrane of CD4^+^ T cells through its key protein myeloperoxidase (MPO) and subsequently recruited interferon-induced transmembrane protein 2 (IFITM2). IFITM2 acted as a DNA receptor that sensed NET-DNA and activated intracellular RAS-associated protein 1B (RAP1B) and its downstream ERK signaling pathway to promote Treg differentiation and function. ENO1 inhibition significantly attenuated NET-induced Treg differentiation and alleviated sepsis in mice. Overall, we demonstrated the role of NETs in sepsis-induced immunosuppression by enhancing Treg differentiation, identified ENO1 as an anchor of NET-MPO, and elucidated the downstream molecular mechanism by which IFITM2-RAP1B-ERK regulates Treg differentiation. These findings improve our understanding of the immunopathogenesis of sepsis and provide potential therapeutic targets for sepsis-induced immunosuppression.

## Abstract 2

NETs-MPO-anchored ENO1 mediates the role of NETs-DNA in promoting Treg differentiation and function in sepsis-induced immunosuppression via a newly identified DNA sensor IFITM2.

## Graphical abstract

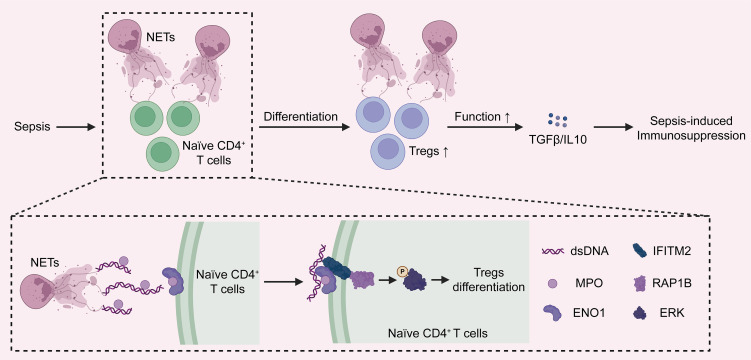

## Introduction

Sepsis is a life-threatening syndrome caused by a dysregulated host response to infection and is one of the leading causes of morbidity and mortality worldwide (1). Pathophysiological studies have demonstrated the critical contribution of immunopathogenesis to sepsis, characterized by hyperinflammation and immunosuppression (2). During sepsis, pro- and antiinflammatory mechanisms interact simultaneously. The proinflammatory response involves the release of proinflammatory mediators as well as the activation of the complement and coagulation systems, among other pathways. Excessive inflammation can cause damage to healthy tissues. Along with the eradication of infectious pathogens by inflammatory responses, antiinflammatory reactions are triggered to control inflammation and facilitate tissue homeostasis. This antiinflammatory response can impair immune cell function through mechanisms such as effector cell apoptosis, T cell exhaustion, and increased expression of suppressor cells. However, in sepsis, antiinflammatory reactions can become unbalanced and contribute to sustained immunosuppression (2). Long-term sepsis-induced immunosuppression increases the susceptibility of patients to secondary infections and poor prognosis (3). Immunotherapies are being developed to reverse immunosuppression, yet therapeutic effects are mixed (4). Studies are needed to elucidate better the complexity and breadth of immune mechanisms involved in sepsis, especially sepsis-induced immunosuppression.

Sepsis-induced immunosuppression is primarily characterized by a reduction of immune effector cells, such as effector T cells (Teffs), DCs, and NK cells (5). This decrease is primarily attributed to the increased number and overactivation of Tregs and is associated with the long-term mortality of septic patients (6). Tregs exert immunosuppressive effects by inhibiting Teff proliferation and function via IL-10 and TGF-β. In addition, Tregs also promote DC apoptosis and dysfunction via cytotoxic T lymphocyte–associated antigen 4 (CTLA4) and inducible T cell costimulator (7–9). These findings suggest that Tregs play a central role in sepsis-induced immunosuppression. Therefore, decoding the underlying regulatory mechanisms governing Treg differentiation and function in sepsis-induced immunosuppression can provide potential targets for immunotherapies for sepsis.

Neutrophil extracellular traps (NETs) are web-like structures released from activated neutrophils that are composed of DNA, histones, and cytotoxic granule–derived proteins such as neutrophil elastase (NE) and myeloperoxidase (MPO) (10). NET production is one of the primary protective responses of neutrophils against infections during the inflammatory phase of sepsis (11), whereas excessive production of NETs further induces the progression of sepsis (12). However, few studies have explored the role of NETs in inducing septic immunosuppression. It has been reported that NETs can induce Treg differentiation through TLR4 and promote the development of liver cancer (13). Therefore, we wondered whether NETs play a role in regulating Treg differentiation and function in sepsis-induced immunosuppression.

In this study, we found that NETs contributed to sepsis-induced immunosuppression by promoting the differentiation and function of Tregs via direct binding to CD4^+^ T cells. Enolase 1 (ENO1), expressed on the surface of CD4^+^ T cells, was identified as the receptor of NETs anchored by MPO. NET-MPO–anchored ENO1 recruited IFN-induced transmembrane protein 2 (IFITM2) to transmit the NET-DNA signal, which subsequently activated the intracellular molecule RAS-associated protein 1 (RAP1) and the downstream ERK signaling pathway to promote the differentiation and function of Tregs. Inhibition of ENO1 decreased Treg differentiation and alleviated septic lethality in mice. Overall, our study reveals a mechanism by which NETs regulate the differentiation and function of Tregs in sepsis-induced immunosuppression.

## Results

### Excessive NETs boosted the differentiation and function of Tregs in sepsis-induced immunosuppression.

**Figure 1:**
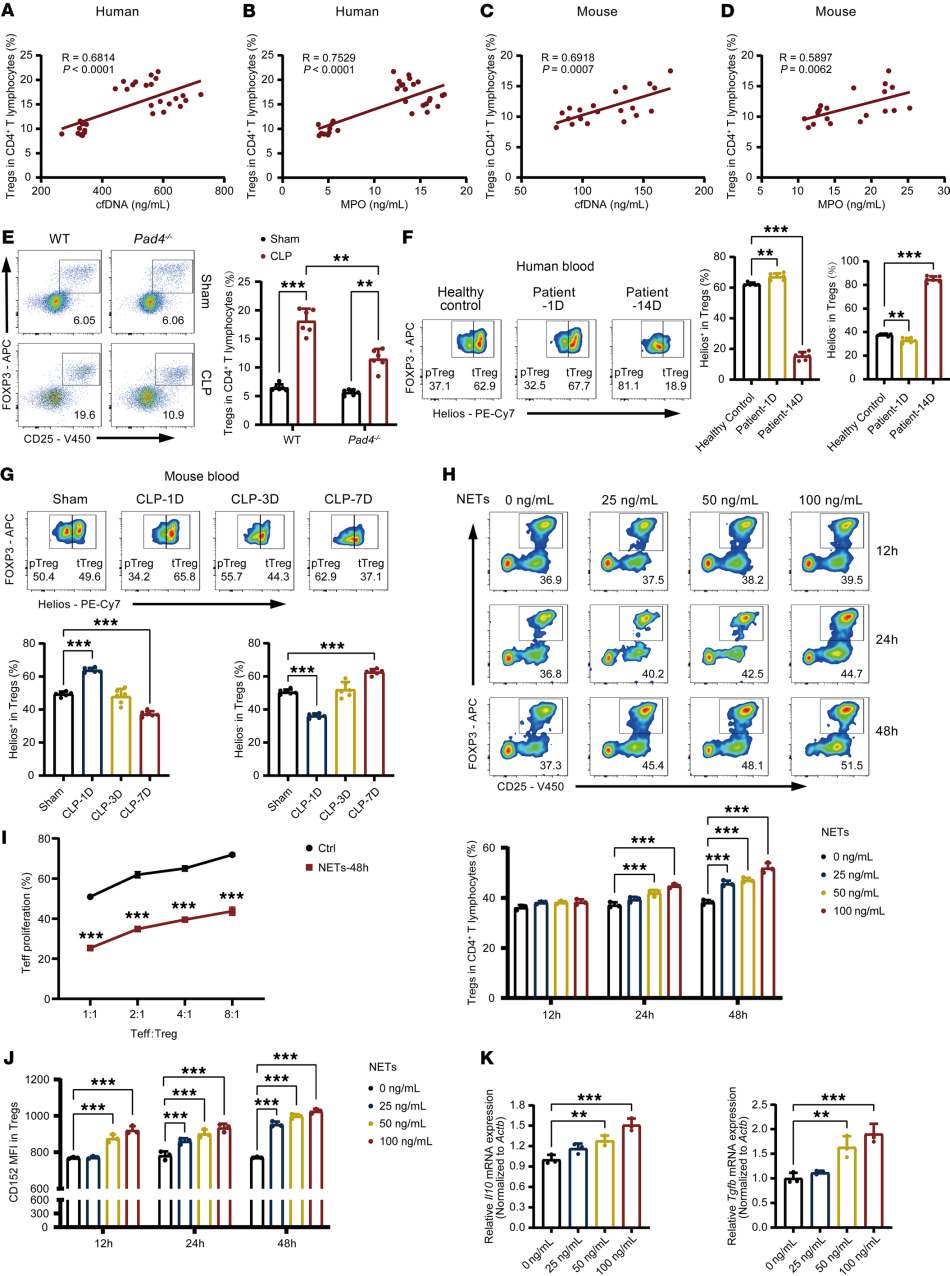
Excessive NETs boosted the differentiation and function of Tregs in sepsis-induced immunosuppression. (**A** and **B**) Correlation analysis was conducted to investigate the potential association between peripheral blood plasma cfDNA (**A**) or MPO (**B**) and the proportion of Tregs in human peripheral blood. Healthy control (*n* = 10), patient-1D (*n* = 10), and patient-14D (*n* = 10). (**C** and **D**) Correlation analysis of the Treg proportion in the spleen with peripheral blood plasma cfDNA (**C**) or MPO (**D**) from mice was conducted. Sham-1D (*n* = 5), CLP-1D (*n* = 5), Sham-7D (*n* = 5), and CLP-7D (*n* = 5). (**E**) The proportion of Tregs was determined in the spleens of WT mice or *Pad4^–/–^* mice 7 days after the CLP or sham procedure by flow cytometry (*n* = 6). (**F** and **G**) The proportion of tTregs and pTregs in the peripheral blood of septic patients and septic mice was determined by flow cytometry (*n* = 6). (**H**) Tregs were differentiated from naive CD4^+^ T cells in vitro after the treatment with NETs (0, 25, 50, and 100 ng/mL; for 12, 24, or 48 hours). Flow cytometry determined the proportion of induced Tregs (*n* = 3). (**I**) The in vitro suppressive function of Tregs on the proliferation of Teffs was assessed, with (NETs-48h) or without (Ctrl) the pretreatment of NETs (100 ng/mL) for 48 hours (*n* = 3). (**J**) The expression level of CD152 in Tregs obtained from in vitro differentiation treated with NETs (0, 25, 50, and 100 ng/mL; for 12, 24, and 48 hours) was determined by flow cytometry (*n* = 3). (**K**) *Il10* and *Tgfb1* mRNA expression in Tregs induced in vitro was measured by reverse transcription–quantitative PCR after treatment with NETs (0, 25, 50, and 100 ng/mL) for 48 hours. Representative data are shown from 3 independent experiments (**E**–**K**). Data are presented as mean ± SD. **P < 0.01, ***P < 0.001. Two-way ANOVA was used for **E** and **H**–**J**, and 1-way ANOVA was used for **F**, **G**, and **K**.

We found an increase in the proportion of Tregs (Supplemental Figure 1A; supplemental material available online with this article; https://doi.org/10.1172/JCI183541DS1) and elevated levels of TGF-β1 and IL-10 (Supplemental Figure 1B) in the peripheral blood of septic patients during the immunosuppressive phase, suggesting a significant enhancement in Treg-mediated immunosuppression. Compared with healthy controls, septic patients exhibited sustained elevated levels of biomarkers of NETs, cell-free DNA (cfDNA), MPO, and the MPO-DNA complex in the peripheral blood plasma (Supplemental Figure 1C). Although the levels of NETs were slightly reduced during the immunosuppressive phase of sepsis compared with the acute phase, they remained elevated (Supplemental Figure 1C). Sepsis patients experienced a rapid increase in NET levels during the acute phase, which is crucial for pathogen clearance. However, during the immunosuppressive phase, the excessive presence of NETs may take on alternative functions. We found that the levels of cfDNA and MPO were positively correlated with the proportion of Tregs in peripheral blood (Figure 1, A and B). These observations in human samples indicated the potential contribution of NETs to the progression of Treg-mediated septic immunosuppression.

A murine model of sepsis induced by cecal ligation and puncture (CLP) (14) was then established (Supplemental Figure 2). We observed that the proportion of Tregs was significantly greater in the peripheral blood and spleens of mice 7 days post-CLP than in those of sham mice or mice 1 day post-CLP (Supplemental Figure 3, A and B), as were the levels of TGF-β1 and IL-10 (Supplemental Figure 3C). In addition, CTLA4 (CD152) expression was elevated in Tregs from mice 7 days post-CLP (Supplemental Figure 3, D–E). Tregs isolated from immunosuppressive phase septic mice demonstrated significantly enhanced suppression of Teff proliferation (Supplemental Figure 3F). These results indicated a Treg-mediated immunosuppressive state in mice 7 days post-CLP, which mimicked the immunosuppressive phase in septic patients.

Consistent with clinical results, the levels of NETs in peripheral blood plasma were sustainedly elevated in septic mice (Supplemental Figure 3G). In addition, the expression of citrullinated histone H3 (CitH3), another critical marker of NETs, was increased in the spleens of CLP mice (Supplemental Figure 3H). The levels of cfDNA and MPO in peripheral blood plasma were positively correlated with the proportion of Tregs in the spleens from mice (Figure 1, C and D). Compared with WT mice, *Pad4^–/–^* mice exhibited a decreased proportion of splenic Tregs during the immunosuppressive phase of sepsis (Figure 1E). These findings indicated a close correlation between elevated NETs and the enhanced proportion of Tregs in sepsis-induced immunosuppression.

Notably, our analyses revealed a significant increase in thymus-derived Tregs (tTregs) in the peripheral blood of septic patients and in both the peripheral blood and spleen of septic mice during the acute phase, followed by a decline during the immunosuppressive phase of sepsis (Figure 1, F and G, and Supplemental Figure 3I). Conversely, peripherally derived Tregs (pTregs) displayed an opposing temporal pattern (Figure 1, F and G, and Supplemental Figure 3I). Treg proliferation was assessed through KI67 staining analysis. In both septic patients and murine models, the frequency of KI67^+^ tTregs in peripheral blood was elevated during the acute phase but diminished during the immunosuppressive phase (Supplemental Figure 4, A and C). Conversely, KI67^+^ pTregs displayed an opposing temporal pattern (Supplemental Figure 4, B and C). Splenic analyses in septic mice revealed that while KI67^+^ tTregs followed the same trend as observed in peripheral circulation, KI67^+^ pTregs were significantly reduced during both disease phases (Supplemental Figure 4D). This may reflect the substantial T cell depletion occurring during immunosuppression, when pTreg generation appears to occur predominantly through induced differentiation rather than proliferative expansion. These results support a model wherein tTreg expansion mediates acute phase immune regulation, while pTreg induction becomes the dominant mechanism during the immunosuppressive phase of sepsis.

We focused our investigation of septic immunosuppression on pTreg differentiation. Peripheral Tregs are derived from naive CD4^+^ T cells induced by specific factors (15). To verify whether NETs play a role in Treg differentiation, an in vitro system of Treg differentiation was established (Supplemental Figure 4, E–G). Treatment of NETs significantly increased the proportion of Tregs differentiated from naive CD4^+^ T cells in a time- and dose-dependent manner (Figure 1H). Furthermore, we observed increased FOXP3 protein expression in NET-treated groups (Supplemental Figure 4, H and I), potentially indicating that NETs may enhance the in vitro differentiation of Tregs. To evaluate the immunosuppressive capacity of NET-activated Tregs, we purified Tregs from splenocytes of healthy mice, followed by in vitro pretreatment with NETs prior to coculture assays. As demonstrated in Figure 1I, NET-pretreated Tregs exhibited enhanced suppression of Teff proliferation across different Teff/Treg ratios, suggesting that NETs potently augment Treg functionality. The levels of CD152 and the expression of *Tgfb1* and *Il10* in NET-treated Tregs were also significantly elevated (Figure 1, J and K). These data suggested that excessive NETs during sepsis-induced immunosuppression promote the differentiation and function of Tregs.

### The NET-DNA-MPO complex was sufficient for regulating Tregs.

**Figure 2:**
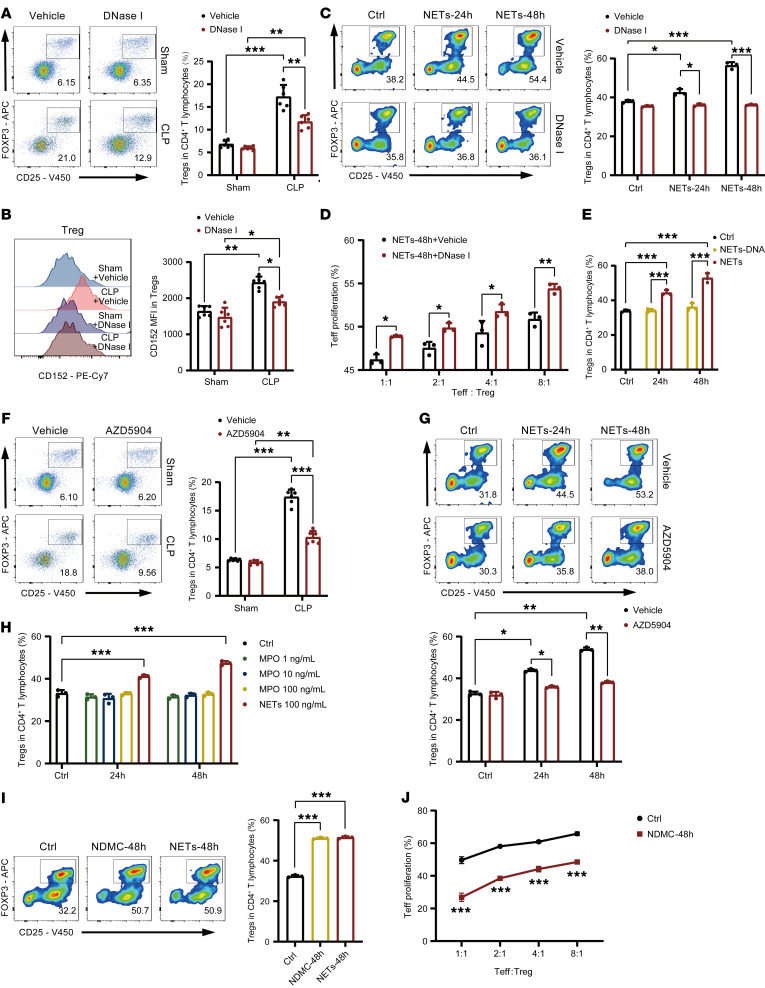
The NDMC was sufficient for regulating Tregs. (**A** and **B**) Mice in the intervention group received i.p. injections of NET-degrading DNase I (5 mg/kg), while vehicle-treated mice received PBS daily from the day before CLP or sham operation until the end of the experiment, 7 days after operation. (**A**) The proportion of Tregs in the spleen was measured by flow cytometry (*n* = 6). (**B**) The expression of CD152 in Tregs from the spleens of CLP or sham mice with or without DNase I (5 mg/kg) was detected by flow cytometry (*n* = 6). (**C**) NET (100 ng/mL), DNase I (2 μg/mL), or vehicle (PBS) was administered to the Treg differentiation system in vitro, and the proportion of induced Tregs was determined by flow cytometry (*n* = 3). (**D**) Suppressive function of Tregs pretreated with NETs (100 ng/mL) and DNase I (2 μg/mL) or vehicle on the proliferation of Teffs was determined (*n* = 3). (**E**) NETs (100 ng/mL) or purified NET-DNA (100 ng/mL) were added during in vitro Treg differentiation, and the proportion of Tregs was measured by flow cytometry (*n* = 3). (**F**) Mice in the intervention group were given an i.p. injection of the selective MPO inhibitor AZD5904 (10 mg/kg) daily from the day before CLP or sham operation, while vehicle-treated mice were given PBS until the end of the experiment on day 7 after surgery. The proportion of Tregs in the spleen was detected by flow cytometry (*n* = 6). (**G**) Treg differentiation in the NET-treated (100 ng/mL) or control group was stimulated with AZD5904 (10 μM) or vehicle. The proportion of Tregs obtained was measured by flow cytometry (*n* = 3). (**H**) Different concentrations of purified MPO (1, 10, and 100 ng/mL) or NETs (100 ng/mL) were added to the in vitro Treg differentiation system. Flow cytometry was used to assess the proportion of Tregs obtained (*n* = 3). (**I**) NETs (100 ng/mL) or NDMC (100 ng/mL) were administered during in vitro Treg differentiation for 48 hours, and the proportion of induced Tregs was determined by flow cytometry (*n* = 3). (**J**) The in vitro suppressive function of Tregs on the proliferation of Teffs was assessed, with or without the pretreatment of NDMC (100 ng/mL) for 48 hours (*n* = 3). Data are representative of 3 independent experiments (**A**–**J**). Data are presented as mean ± SD. *P < 0.05, **P < 0.01, ***P < 0.001. Two-way ANOVA was used for **A**–**H** and **J**, and 1-way ANOVA was used for **I**.

cfDNA, the significant component of NETs, plays a central role in mediating the biological effects of NETs via binding to DNA sensors (16). When NET-DNA was degraded by DNase I (Supplemental Figure 5A), the proportion of Tregs dramatically decreased in CLP-induced septic mice (Figure 2A). DNase I administration also reduced the expression of CD152 in Tregs (Figure 2B) and the levels of TGF-β1 and IL-10 in peripheral blood plasma (Supplemental Figure 5B). Consistently, the ability of NETs to promote the in vitro differentiation of Tregs was also significantly attenuated (Figure 2C and Supplemental Figure 5, C and D). Functionally, DNase I treatment significantly attenuated the suppressive effect of Tregs on Teff proliferation (Figure 2D), reduced the level of CD152 (Supplemental Figure 5E), and decreased the mRNA expression of *Tgfb1* and *Il10* (Supplemental Figure 5F). However, purified NET-DNA exhibited little effect on promoting the differentiation of Tregs (Figure 2E), suggesting a necessary but not sufficient role for NET-DNA in inducing Treg differentiation and function.

MPO, a neutrophil-specific protein essential for forming NETs, has been shown to directly or indirectly regulate immune responses in both homeostatic and disease states (17–19). We found that the administration of the selective MPO inhibitor AZD5904 led to a significant decrease in the proportion of Tregs in septic mice (Figure 2F). The effect of NETs on promoting in vitro Treg differentiation was also attenuated by AZD5904 (Figure 2G). However, although the level of MPO was unchanged upon DNase I treatment (Supplemental Figure 5G), the ability of NETs to promote Treg differentiation was abolished. Similarly, purified MPO did not affect the differentiation of Tregs in vitro (Figure 2H). These results suggested that MPO was another necessary but not sufficient component of NET regulation of Tregs.

We also investigated the involvement of other components of NETs, such as NE and CitH3, in Treg regulation; however, our data did not reveal a marked role for either NE or CitH3 in this context (data not shown). To investigate whether the combination of cfDNA and MPO was sufficient for mediating the effect of NETs on Tregs, we constructed a NET-DNA-MPO complex (NDMC) by coincubating purified NET-DNA with MPO and investigated its role in regulating Tregs (Supplemental Figure 5, H and I). As expected, NDMC significantly enhanced Treg differentiation, similar to the effect of NETs (Figure 2I). Moreover, the suppressive effect of Tregs on Teff proliferation was augmented by NDMC (Figure 2J). The level of CD152 in NDMC-treated Tregs was also elevated (Supplemental Figure 5J), accompanied by increased mRNA expression of *Tgfb1* and *Il10* (Supplemental Figure 5K). Furthermore, our studies demonstrated that NDMC could induce partial CD4^+^ T cell differentiation even in the absence of canonical Treg induction factors, with enhanced effects observed when combined with the complete Treg differentiation system (Supplemental Figure 6A). Notably, NDMC showed no significant impact on Th1, Th2, or Th17 cell differentiation, regardless of Treg induction conditions (Supplemental Figure 6B). Further investigation using established in vitro polarization protocols confirmed that NDMC did not alter Th1, Th2, or Th17 subset specification (Supplemental Figure 6C). These observations collectively indicated that the cfDNA and MPO in NETs co-mediated the regulatory effect of NETs on Tregs.

### Cell surface ENO1 interacted with MPO to navigate the effects of NDMC on Tregs.

**Figure 3:**
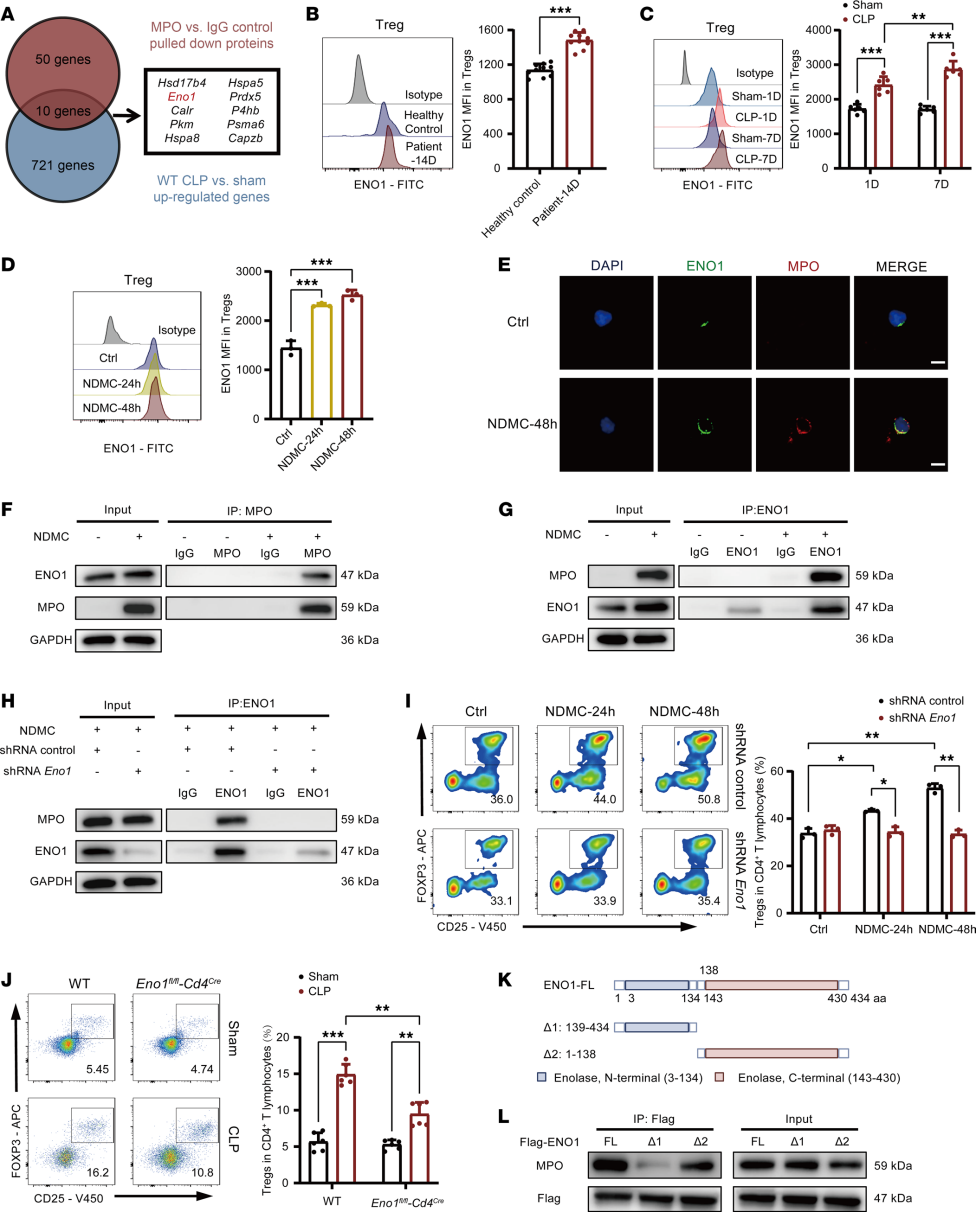
Cell surface ENO1 interacted with MPO to navigate the effects of NDMC on Tregs. (**A**) Proteins interacting with MPO in the lysate of in vitro–induced Tregs treated with 500 ng/mL NETs were analyzed by nanoLC-MS/MS. The upregulated genes in CD4^+^ T cells collected from the spleens of CLP-operated mice versus sham-operated mice that overlapped with the purification of MPO-treated versus IgG control–treated pulled-down proteins are shown in the Venn diagram. (**B**) The expression of ENO1 on Tregs in the peripheral blood of healthy controls and patients during the immunosuppressive phase of sepsis was determined by flow cytometry (*n* = 10). (**C**) The expression of ENO1 on Tregs in the spleens of CLP or sham mice 1 or 7 days after operation was determined by flow cytometry (*n* = 6). (**D**) The expression of ENO1 on Tregs induced in vitro under NDMC treatment (100 ng/mL) for 24 or 48 hours was determined by flow cytometry (*n* = 3). (**E**) Immunofluorescence staining detected the colocalization of MPO and ENO1 on the cell membrane of induced Tregs with NDMC (100 ng/mL) or vehicle. Scale bars: 5 μm. Green, ENO1; red, MPO. (**F**) Immunoblot analysis of ENO1 immunoprecipitated with MPO from naive CD4^+^ T cells under Treg differentiation conditions treated with NDMC (500 ng/mL) or vehicle. (**G**) Immunoblot analysis of MPO immunoprecipitated with ENO1 from naive CD4^+^ T cells under Treg differentiation conditions with or without NDMC treatment (500 ng/mL). (**H**) Lentivirus-shRNA (LV-shRNA) *Eno1* was used to knock down the *Eno1* gene in naive CD4^+^ T cells. Immunoblot analysis of MPO immunoprecipitated with ENO1 from NDMC-treated (500 ng/mL) naive CD4^+^ T cells under Treg differentiation conditions treated with LV-shRNA control or LV-shRNA *Eno1*. (**I**) Naive CD4^+^ T cells transfected with LV-shRNA *Eno1* or LV-shRNA control were induced to differentiate into Tregs in vitro with or without NDMC treatment (100 ng/mL). Flow cytometry was used to evaluate the proportion of Tregs (*n* = 3). (**J**) The proportion of Tregs was analyzed in the spleens of WT mice or *Eno1^fl/fl^ Cd4^Cre^* mice 7 days after the CLP or sham procedure by flow cytometry (*n* = 6). (**K**) Flag-tagged full-length (FL) ENO1 or one of its truncated mutants (Δ1 to Δ2) was transfected into HEK293T cells. (**L**) Immunoblot analysis was conducted for MPO immunoprecipitated with beads coated with a Flag antibody. Representative data are shown from 3 independent experiments (**B**–**J** and **L**). Data are represented as mean ± SD. *P < 0.05, **P < 0.01, ***P < 0.001. Two-tailed Student’s *t* test was used for **B**; 2-way ANOVA was used for **C**, **I**, and **J**; and 1-way ANOVA was used for **D**.

We further investigated how NETs recognized and regulated Tregs during septic immunosuppression. In the in vitro system of Treg differentiation, cfDNA of NETs and NDMC accumulated around CD4^+^ T cells, while purified NET-DNA remained free in the culture medium after incubation, indicating that MPO served as an anchor protein for tethering cfDNA to CD4^+^ T cells (Supplemental Figure 7A). To identify the potential receptor of MPO on the surface of CD4^+^ T cells, 2 experiments were conducted: first, nanoflow liquid chromatography–tandem mass spectrometry (nanoLC-MS/MS**)** was employed to analyze proteins interacting with MPO in Tregs induced in vitro; second, RNA-Seq was performed to screen the differentially expressed genes encoding membrane proteins in CD4^+^ T cells collected from the spleens of CLP and sham mice 7 days after operation (Supplemental Figure 7B). The pulled-down MPO target proteins that overlapped with the upregulated genes in CD4^+^ T cells from septic mice are highlighted in Figure 3A. Among these proteins, ENO1 may be the most promising receptor of NET-MPO on CD4^+^ T cells and is involved in sepsis-induced immunosuppression.

ENO1 is a multifunctional protein that acts as a cytoplasmic glycolytic enzyme and a receptor for plasminogen on the cell surface (20, 21). Considering that NETs or NDMC were found around CD4^+^ T cells, ENO1 on the cell surface was the most likely form to which NET-MPO bound. To confirm our hypothesis, we initially assessed the expression pattern of ENO1 on the surface of Tregs. Data showed that there was a significantly elevated level of ENO1 on the surface of Tregs derived from sepsis patients compared with those derived from healthy controls (Figure 3B). A similar phenomenon was found in Tregs obtained from CLP mice (Figure 3C) and in vitro–induced Tregs treated with NDMC (Figure 3D). Furthermore, we extracted cell membrane proteins from in vitro–induced Tregs to quantify the level of ENO1 and detected a pronounced increase in ENO1 expression in response to NDMC treatment (Supplemental Figure 7C). Interestingly, upon treatment with DNase I, the NDMC-induced elevation in ENO1 expression on the surface of Tregs was abolished (Supplemental Figure 7D), and neither the addition of purified NET-DNA nor the addition of purified MPO altered the expression of ENO1 on Tregs (Supplemental Figure 7, E and F). Notably, NDMC treatment did not significantly alter ENO1 expression levels on Th1, Th2, or Th17 cells (Supplemental Figure 6D). Consistent with this observation, sepsis-induced immunosuppression in murine models also failed to modify ENO1 expression in these helper T cell subsets (Supplemental Figure 6, E–G). These data suggested that NDMC induced upregulation of ENO1 on the surface of Tregs, which was related to the regulation of Tregs.

As expected, colocalization of MPO and ENO1 was observed on the cell membrane of induced Tregs treated with NDMC (Figure 3E and Supplemental Figure 7G). The interaction of NET-MPO with cell surface ENO1 was further confirmed by IP (Figure 3, F and G). The expression of MPO in induced Tregs without adding NDMC was almost undetectable (Supplemental Figure 7H), suggesting that the MPO bound to ENO1 on the CD4^+^ T cell surface was directly derived from NDMC. In addition, knockdown of the *Eno1* gene in naive CD4^+^ T cells inhibited the formation of the MPO-ENO1 complex (Figure 3H and Supplemental Figure 7, I and J) and significantly attenuated the differentiation of Tregs by NDMC (Figure 3I and Supplemental Figure 7, K and L). Compared with WT mice, *Eno1^fl/fl^ Cd4^Cre^* mice exhibited a decreased proportion of Tregs in the immunosuppressive phase of sepsis (Figure 3J and Supplemental Figure 7M) and a higher survival rate following the secondary *Pseudomonas aeruginosa* challenge (Supplemental Figure 7N).

To explore how ENO1 interacts with NET-MPO, we constructed 2 truncated mutants of ENO1 to identify the specific region involved in this interaction. Co-IP analysis in HEK293T cells revealed that the deletion of C-terminal amino acids (Δ1, 139–434 amino acids) of ENO1 eliminated the interaction between ENO1 and NET-MPO (Figure 3, K and L), suggesting that this domain was responsible for the interaction. These findings demonstrated that NET-MPO interacted with naive CD4^+^ T cells by recognizing and binding to ENO1 on the cell surface, thereby promoting the accumulation of NET/NDMC around CD4^+^ T cells. Moreover, the C-terminal amino acid 139–434 domain of ENO1 was the binding site of NET-MPO.

### MPO-ENO1 transmitted stimulatory signals for NET-DNA through upregulating IFITM2.

**Figure 4:**
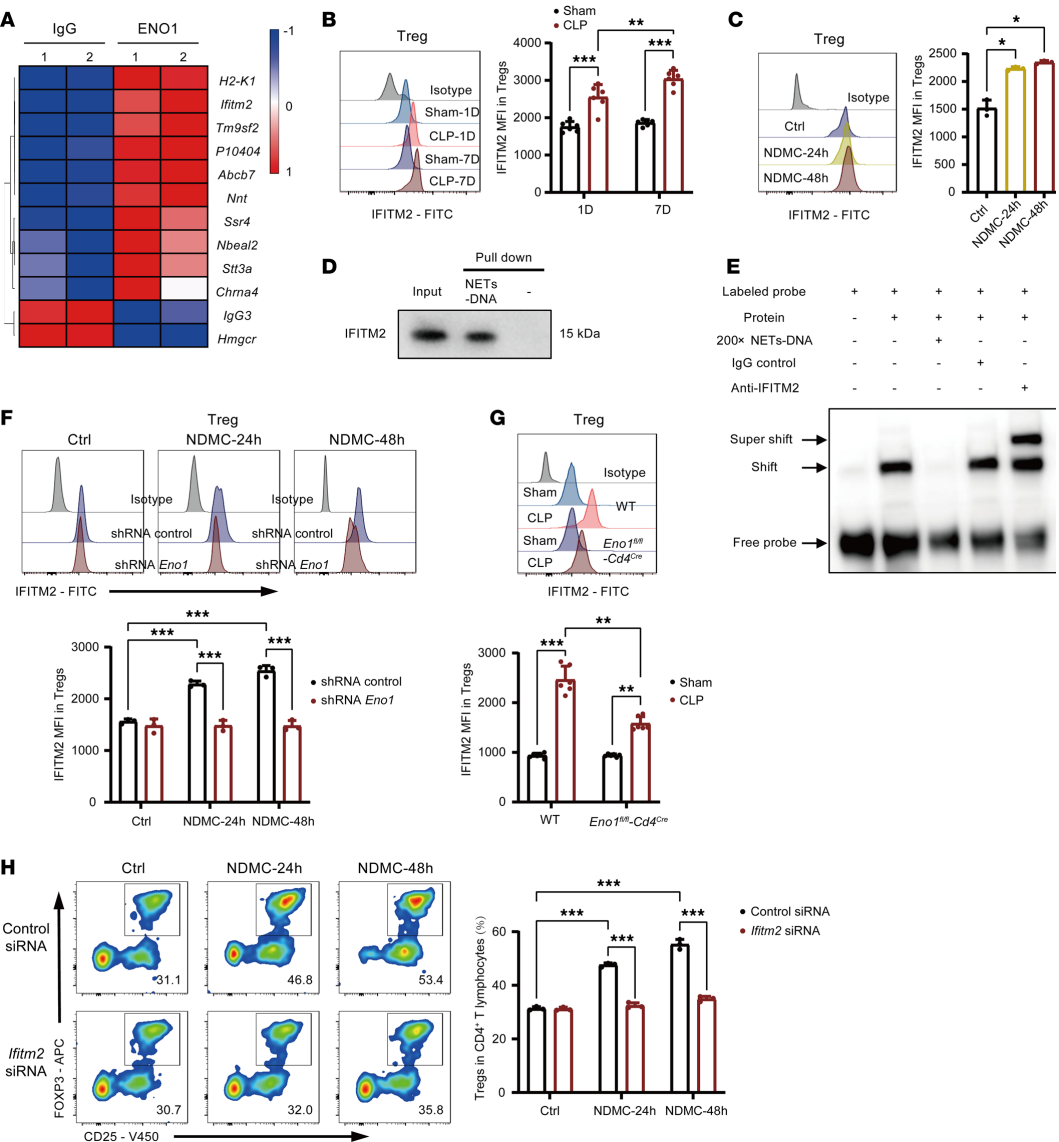
MPO-ENO1 transmitted stimulatory signals for NET-DNA through upregulating IFITM2. (**A**) ENO1 co-IP experiments were performed using lysates from in vitro–induced Tregs treated with NDMC (500 ng/mL), and nanoLC-MS/MS was used to identify the proteins bound to ENO1. ENO1-binding membrane proteins are shown in the heatmap (*n* = 2). (**B**) The expression of IFITM2 on Tregs in the spleens of mice 1 or 7 days after the CLP or sham procedure (*n* = 6). (**C**) The expression of IFITM2 on Tregs induced in vitro with or without NDMC treatment (100 ng/mL) was determined by flow cytometry (*n* = 3). (**D**) Immunoblot analysis of the lysates of in vitro–induced Tregs pulled down by biotinylated NET-DNA (500 ng) and detected with an IFITM2 antibody. (**E**) The membrane proteins of in vitro–induced Tregs (10 μg) and the biotinylated NET-DNA (1 ng) were incubated with or without a 200-fold excess of unbiotinylated NET-DNA, an IFITM2 antibody, or IgG control. EMSA demonstrated that the IFITM2 antibody supershifted the binding of NET-DNA to IFITM2. (**F**) Naive CD4^+^ T cells were transfected with LV-shRNA control or LV-shRNA *Eno1* and then induced to Tregs in vitro with or without NDMC (100 ng/mL). The expression of IFITM2 on induced Tregs was determined by flow cytometry (*n* = 3). (**G**) IFITM2 expression of Tregs in the spleens of WT mice or *Eno1^fl/fl^ Cd4^Cre^* mice 7 days after the CLP or sham procedure was determined by flow cytometry (*n* = 6). (**H**) Naive CD4^+^ T cells were transfected with control siRNA or *Ifitm2* siRNA and then induced to differentiate into Tregs in vitro with or without the addition of NDMC (100 ng/mL). Flow cytometry was used to assess the proportion of Tregs (*n* = 3). Representative data are shown from 3 independent experiments (**B**–**H**). Data are presented as mean ± SD. *P < 0.05, **P < 0.01, ***P < 0.001. Two-way ANOVA was used for **B** and **F**-**H**, and 1-way ANOVA was used for **C**.

After NET-MPO recognizes and binds to ENO1, intracellular signaling pathways need to be triggered to initiate the regulation of Tregs. Since ENO1 lacks a transmembrane region, as shown by the transmembrane helix prediction algorithm (Supplemental Figure 8A), we speculated that there might be another specific transmembrane protein bound to ENO1 on naive CD4^+^ T cells that mediated the effect of NDMC on Tregs. We analyzed ENO1-binding membrane proteins in naive CD4^+^ T cells treated with NDMC and identified several candidates (Figure 4A). IFITM2 was significantly upregulated in the splenic Tregs of septic mice and in vitro–induced Tregs treated with NDMC (Figure 4, B and C). Consistent results were observed for the expression of IFITM2, aligning with the expression trend of ENO1 (Supplemental Figure 8B). Remarkably, the effects of CLP modeling and NDMC stimulation on the upregulation of IFITM2 expression in Tregs were significantly attenuated after DNase I intervention (Supplemental Figure 8, C and D), suggesting its dependence on NDMC. Moreover, the direct interaction of IFITM2 with ENO1 on the cell membrane was verified by immunofluorescence (Supplemental Figure 8, E and F) and IP (Supplemental Figure 8G). Notably, NET-MPO-ENO1-IFITM2 complex formation was detectable within 24 hours of NET stimulation (Supplemental Figure 8H), suggesting a potential role for NETs in initiating and promoting Treg differentiation. Finally, IFITM2 interacted with NET-DNA (Figure 4, D and E), indicating that IFITM2 acted as a DNA sensor to transmit the stimulatory signals of NDMC.

To further confirm the role of IFITM2 in mediating NDMC regulation on Tregs, we knocked down the *Eno1* or *Ifitm2* gene in naive CD4^+^ T cells. Compared with the control group, the expression of IFITM2 in NDMC-stimulated Tregs was significantly reduced upon *Eno1* knockdown (Figure 4F). The expression of IFITM2 in splenic Tregs was also reduced in *Eno1^fl/fl^ Cd4^Cre^* mice during septic immunosuppression (Figure 4G), suggesting that the upregulation of IFITM2 in sepsis is related to ENO1. Intriguingly, knocking down the *Ifitm2* gene in naive CD4^+^ T cells significantly attenuated the ability of NDMC to induce Treg differentiation (Figure 4H and Supplemental Figure 8I), while the knockdown of the *Ifitm2* gene did not affect the expression of ENO1 in NDMC-treated Tregs (Supplemental Figure 8J). These data suggested that NET-MPO–anchored ENO1 on the cell surface further bound to the transmembrane protein IFITM2 for intracellular signal transduction, which promoted Treg differentiation and function and participated in sepsis-induced immunosuppression.

### IFITM2 interacted with RAP1 and facilitated NDMC-promoted Treg differentiation and function.

**Figure 5:**
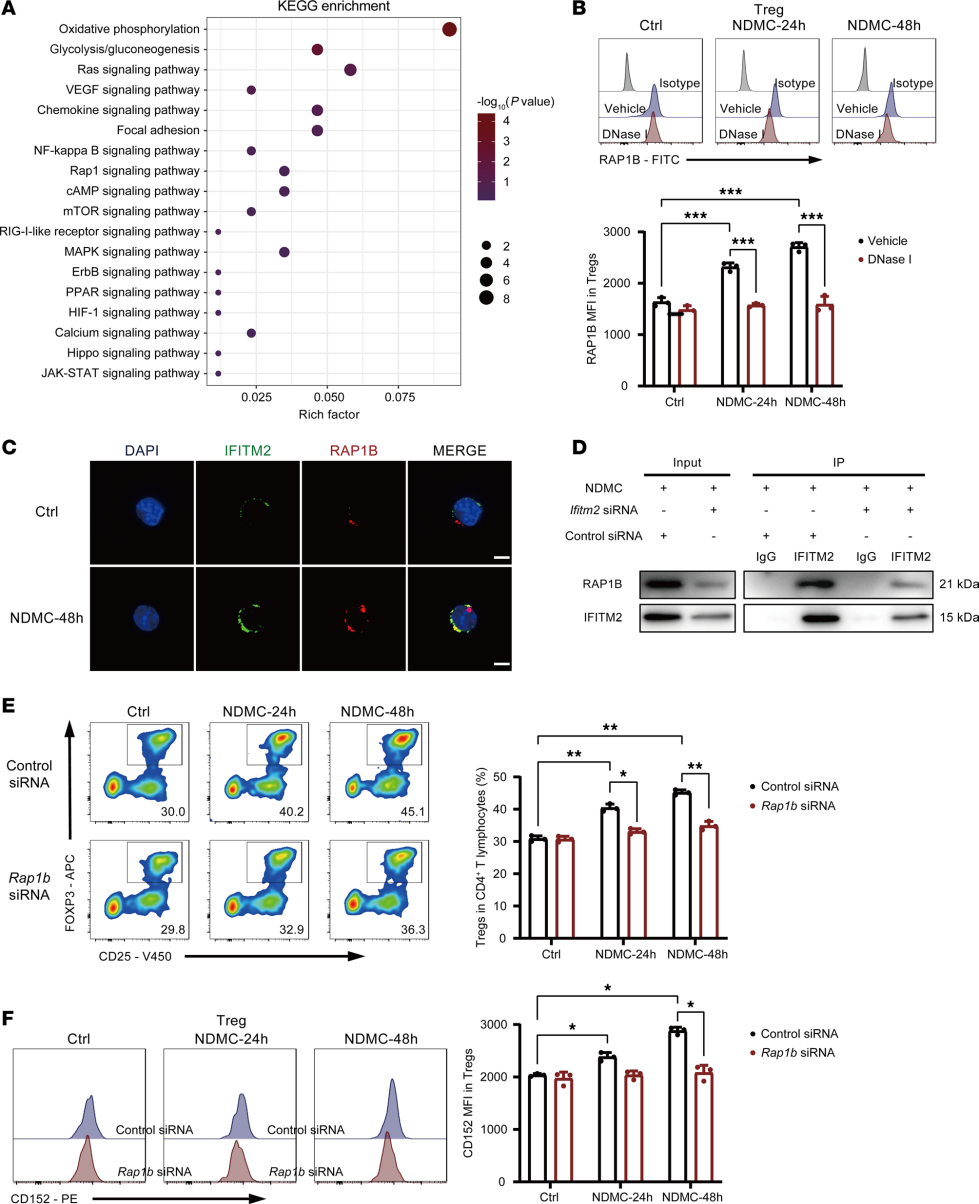
IFITM2 interacted with RAP1 and facilitated NDMC-promoting Treg differentiation and function. (**A**) KEGG enrichment analysis was conducted based on IFITM2-binding cytoplasm proteins in naive CD4^+^ T cells under Treg differentiation conditions treated with NDMC (500 ng/mL). Enriched signaling pathways are shown. (**B**) The expression of RAP1B in Tregs induced in vitro with or without NDMC (100 ng/mL) or DNase I (2 μg/mL) treatment was determined by flow cytometry (*n* = 3). (**C**) The colocalization of RAP1B and IFITM2 in naive CD4^+^ T cells under Treg differentiation conditions treated with NDMC (100 ng/mL) or vehicle was observed by immunofluorescence staining. Scale bars: 5 μm. Green, IFITM2; red, RAP1B. (**D**) Naive CD4^+^ T cells were transfected with control siRNA or *Ifitm2* siRNA and then treated with or without NDMC (500 ng/mL) under Treg differentiation conditions. RAP1B immunoprecipitated with IFITM2 was determined by immunoblot. (**E** and **F**) Naive CD4^+^ T cells were transfected with control siRNA or *Rap1b* siRNA and then induced to differentiate into Tregs in vitro via treatment with NDMC (100 ng/mL) or vehicle. (**E**) Flow cytometry assessed the proportion of Tregs (*n* = 3). (**F**) The expression of CD152 in induced Tregs was determined by flow cytometry (*n* = 3). Data shown are representative of 3 independent experiments (**B**–**F**) and are presented as mean ± SD. *P < 0.05, **P < 0.01, ***P < 0.001. Two-way ANOVA was used for **B**, **E**, and **F**.

To unravel the underlying intracellular signaling pathways of NDMC on Tregs, we performed Kyoto Encyclopedia of Genes and Genomes (KEGG) pathway analysis of IFITM2-related cytoplasm proteins and found that the Ras signaling pathway was significantly enriched (Figure 5A). Meanwhile, RAP1B was an ENO1-IFITM2–binding cytoplasmic protein in CD4^+^ T cells (Supplemental Figure 9A). RAP1 has been shown to promote the differentiation of naive CD4^+^ T cells into Tregs while inhibiting their differentiation into Th17 cells in spontaneous colitis (22, 23), indicating its potential involvement in Treg regulation. Here, we found that RAP1B expression was upregulated in Tregs upon NDMC treatment, and this upregulation was reversed by adding DNase I (Figure 5B).

We observed the colocalization of RAP1B and IFITM2 in CD4^+^ T cells after NDMC stimulation (Figure 5C). However, after silencing of the *Ifitm2* gene in naive CD4^+^ T cells, the pull-down of RAP1B by IFITM2 was attenuated (Figure 5D and Supplemental Figure 9B). Moreover, the expression of RAP1B in splenic Tregs from *Eno1^fl/fl^ Cd4^Cre^* mice during the immunosuppressive phase of sepsis and in NDMC-induced Tregs upon *Eno1* or *lfitm2* knockdown was significantly reduced (Supplemental Figure 9, C–E). Hence, these data suggested that IFITM2 recruited RAP1B. In addition, silencing of the *Rap1b* gene in naive CD4^+^ T cells significantly attenuated the NDMC-mediated increase in Treg differentiation (Figure 5E and Supplemental Figure 9F) as well as the NDMC-induced upregulation of CD152 in Tregs (Figure 5F). Unsurprisingly, it had no significant effect on the expression of ENO1 in Tregs (Supplemental Figure 9G). These data suggested that NET-MPO recognized and bound to the transmembrane complex ENO1-IFITM2, which then recruited RAP1B to regulate Treg differentiation.

### NDMC activated the ERK pathway via RAP1B to promote Treg differentiation and function.

**Figure 6:**
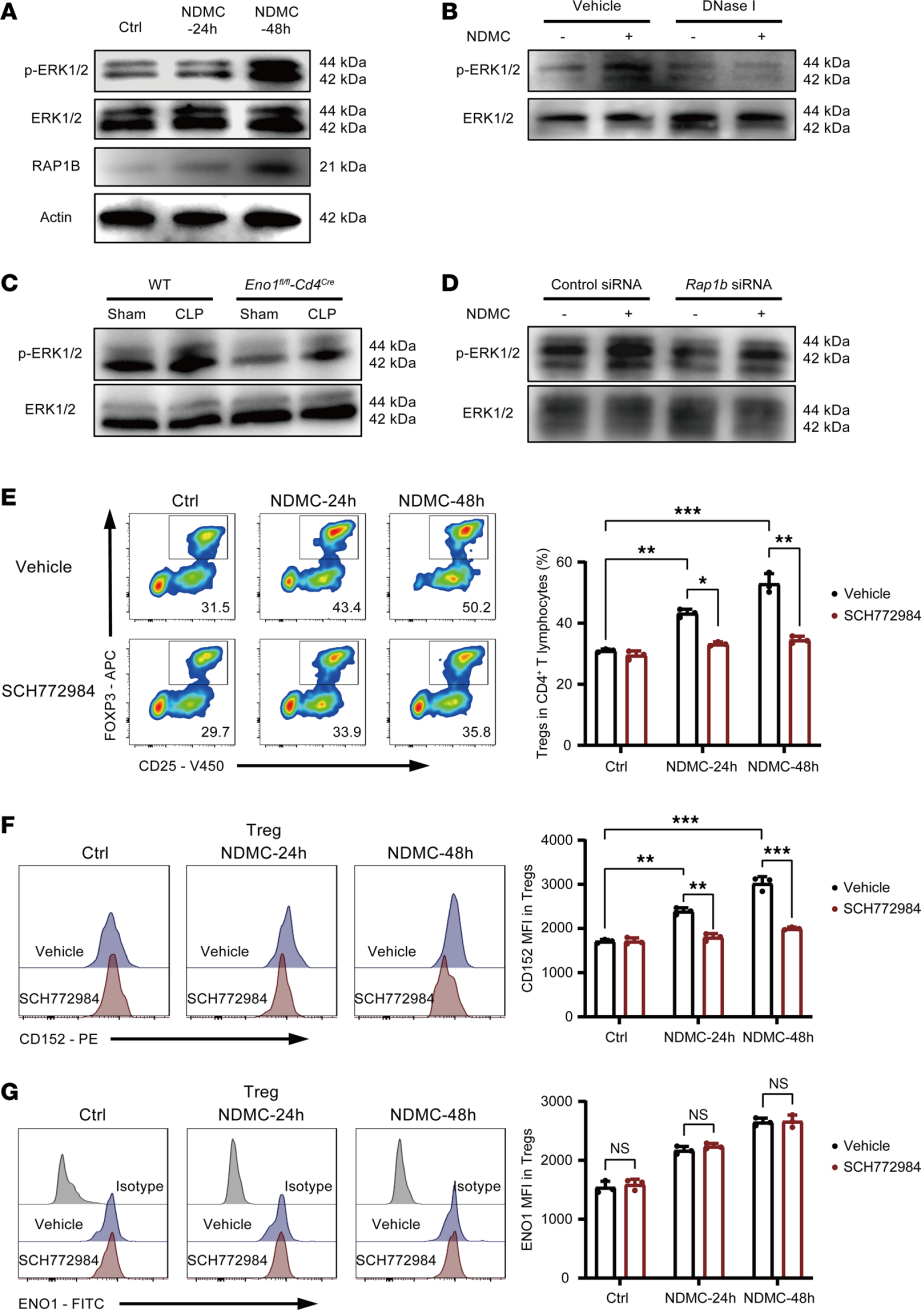
NDMC activated the ERK pathway via RAP1B to promote Treg differentiation and function. (**A**) Naive CD4^+^ T cells were treated with NDMC (100 ng/mL) or vehicle under Treg differentiation conditions. The protein expression levels of RAP1B, ERK1/2, and p-ERK1/2 were then determined by Western blotting. (**B**) RAP1B, ERK1/2, and p-ERK1/2 protein expression in naive CD4^+^ T cells under Treg differentiation conditions with or without NDMC (100 ng/mL) or DNase I (2 μg/mL) treatment was evaluated by Western blotting. (**C**) ERK1/2 and p-ERK1/2 expression in Tregs from the spleens of WT mice or *Eno1^fl/fl^ Cd4^Cre^* mice 7 days after the CLP or sham procedure was determined by Western blotting. (**D**) Naive CD4^+^ T cells were transfected with *Rap1b* siRNA or control siRNA and then induced to differentiate into Tregs in the presence or absence of NDMC (100 ng/mL). The protein expression levels of RAP1B, ERK1/2, and p-ERK1/2 were then determined by Western blotting. (**E**–**G**) The ERK inhibitor SCH772984 (300nM) was added to the in vitro Treg differentiation cultures with or without NDMC (100 ng/mL). (**E**) The proportion of induced Tregs was assessed by flow cytometry (*n* = 3). (**F**) The expression of CD152 in Tregs was determined by flow cytometry (*n* = 3). (**G**) The expression of ENO1 on Tregs was determined by flow cytometry (*n* = 3). Data shown are representative of 3 independent experiments (**A**–**G**) and are presented as mean ± SD. *P < 0.05, **P < 0.01, ***P < 0.001. Two-way ANOVA was used for **E**–**G**.

Activated RAP1B directly activates the ERK signaling pathway (24, 25), which has also been shown to determine the viability and expansion of Tregs (26). We observed that the increased expression of RAP1B in NDMC-stimulated naive CD4^+^ T cells was accompanied by a notable activation of the ERK1/2 signaling pathway (Figure 6A). However, the activation of ERK1/2 was inhibited in DNase I–treated naive CD4^+^ T cells, in splenic Tregs from *Eno1^fl/fl^ Cd4^Cre^* mice, and in naive CD4^+^ T cells upon *Rap1b* knockdown, consistent with the trend of RAP1B protein expression (Figure 6, B–D). After inhibiting the activation of the ERK signaling pathway with the competitive ERK inhibitor SCH772984 in vitro, we found that blocking ERK1/2 significantly attenuated the ability of NDMC to promote Treg differentiation (Figure 6E) and upregulate the expression of CD152 (Figure 6F) but did not affect the expression of ENO1 on Tregs (Figure 6G). These data suggested that RAP1-activated ERK1/2 was the intracellular signaling pathway mediating the effect of NDMC on Tregs.

### Inhibition of ENO1 decreased Treg differentiation and alleviated septic lethality in mice.

**Figure 7:**
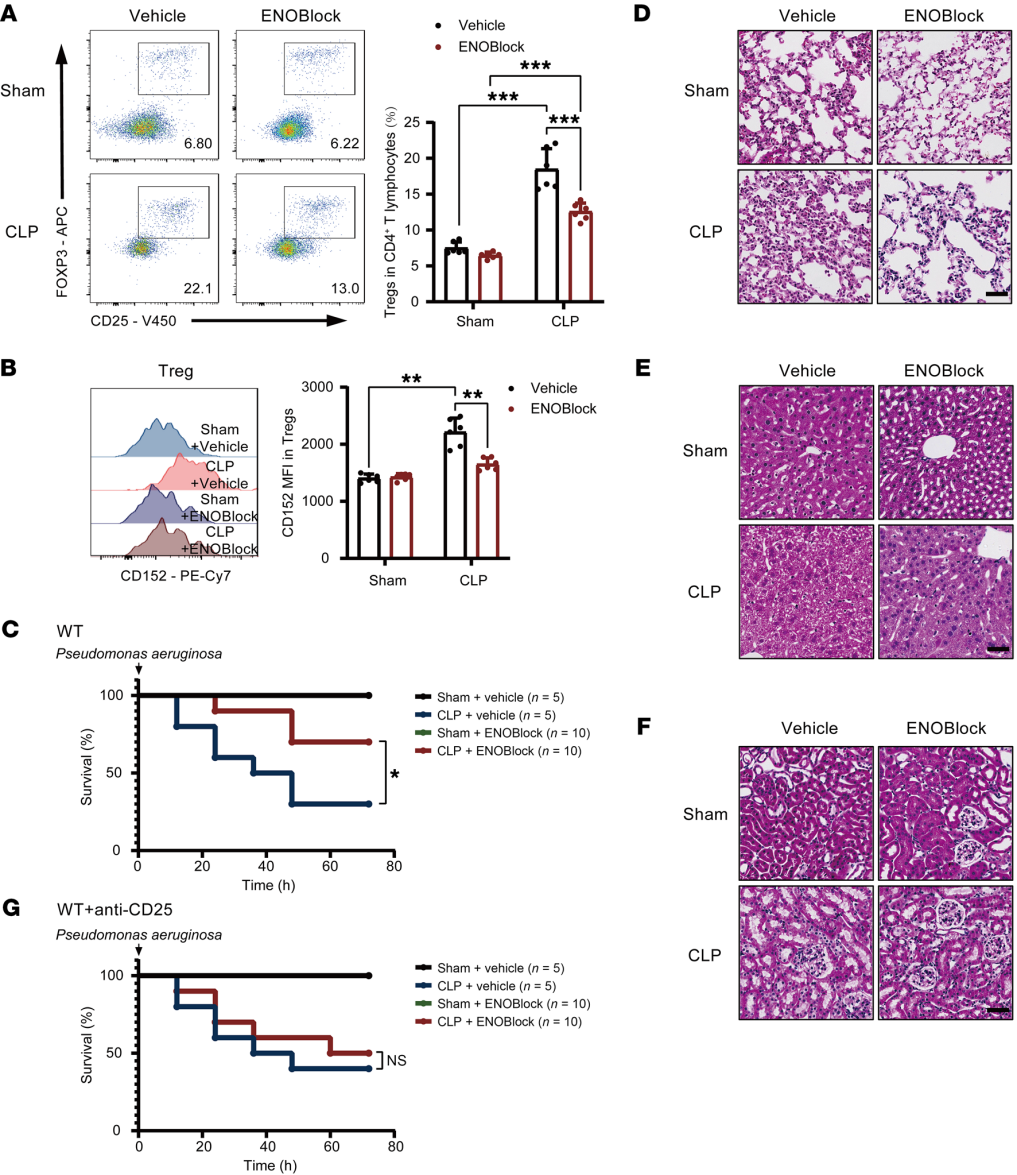
Inhibition of ENO1 decreased Treg differentiation and alleviated septic lethality in mice. To evaluate the effect of ENO1 inhibition on the development of sepsis-induced immunosuppression, mice were i.p. injected with ENOBlock (10 mg/kg). Vehicle-treated mice received solvent every other day from 3 days after surgery until the end of the experiment. The murine spleen, lung, liver, and kidney were collected for further experiments. (**A**) The proportion of Tregs in the spleens of mice was determined by flow cytometry (*n* = 6). (**B**) The expression of CD152 in Tregs in the spleens of mice was determined by flow cytometry (*n* = 6). (**C**) Kaplan-Meier survival analysis was performed for all experimental groups over the 72 hour period after *P*. *aeruginosa* challenge, administered 7 days post-CLP or after sham operation. Sham + vehicle (*n* = 5), CLP + vehicle (*n* = 10), Sham + ENOBlock (*n* = 5), and CLP + ENOBlock (*n* = 10). (**D**–**F**) Mice in the different groups received *P*. *aeruginosa* intranasally on the seventh day after operation. H&E staining was conducted to assess lung injury (**D**), liver injury (**E**), and kidney injury (**F**) 3 days after inhalation. Scale bars: 50 μm. (**G**) Kaplan-Meier survival analysis was performed for CLP and sham-operated mice over 72 hours following *P*. *aeruginosa* challenge (administered 7 days after surgery), with Treg depletion via anti-CD25 mAb (200 μg/mouse) 24 hours prior to inhalation. Sham + vehicle (*n* = 5), CLP + vehicle (*n* = 10), Sham + ENOBlock (*n* = 5), and CLP + ENOBlock (*n* = 10). Representative data are shown from 3 independent experiments (**A**–**G**). Data are presented as mean ± SD. *P < 0.05, **P < 0.01, ***P < 0.001. Two-way ANOVA was used for **A** and **B**, and the log-rank test was used for **C** and **G**.

To explore the potential of ENO1 inhibition in alleviating sepsis, an ENO1 inhibitor (ENOBlock) that is directly bound to ENO1 (27, 28) was used to block the interaction of NET-MPO and ENO1, thereby hampering the recognition of ENO1 by NETs during sepsis. Unsurprisingly, the increase in the expression of ENO1 in the splenic Tregs of septic mice was reversed in mice administered with ENOBlock (Supplemental Figure 10A). Moreover, the proportion of Tregs in the spleens was reduced upon ENO1 inhibition (Figure 7A), as well as the expression of CD152 in Tregs (Figure 7B). In addition, ENOBlock intervention significantly improved the survival rate of septic mice subjected to a secondary infection of *P*. *aeruginosa* (Figure 7C) and ameliorated multiorgan damage (Figure 7, D–F). Treg depletion abolished ENOBlock’s protective effect on mortality reduction in septic mice, demonstrating that ENOBlock exerts its survival benefit by specifically suppressing Treg differentiation and function during sepsis-induced immunosuppression (Figure 7G and Supplemental Figure 10, B and C). These results further confirmed the critical role of ENO1 in NET-mediated Treg regulation and sepsis and, more importantly, suggested the possibility of targeting ENO1 to alleviate the progression of sepsis-induced immunosuppression.

## Discussion

To maintain homeostasis, the hyperinflammatory phase of sepsis, which protects the host against infection, is always simultaneously accompanied by an antiinflammatory and immunosuppressive counterresponse to prevent deleterious hyperinflammation (29). However, when this compensatory mechanism is uncontrolled, it can lead to sepsis-induced immunosuppression status (3).

The role of NETs in sepsis-induced immunosuppression is more complex. When released by neutrophils via NETosis, the primary role of NETs is to clear invading pathogens. Nevertheless, increasing evidence suggests that pronounced elevation in NET formation is closely associated with poor prognosis (30). Our previous study revealed that the level of NETs in patients with sepsis was closely related to the survival rate. Although the role of NETs in sepsis-induced immunosuppression is still unclear, there are hints from studies showing the positive regulatory effect of NETs on suppressive immune cells and cancer metastasis (16, 31, 32). Accordingly, our study provides preliminary clinical evidence for the association between NETs and sepsis-induced immunosuppression. Furthermore, NETs exert their function by promoting the differentiation and activity of Tregs.

Despite abundant studies revealing the multifunctionality of NETs, the underlying mechanisms are poorly understood. NET-DNA is closely associated with the function of NETs, such as NET-mediated pro-metastasis in cancer (33). In addition to NET-DNA, studies on the noncanonical functions of NET-MPO have presented intriguing findings (19). These findings indicate that NETs can function by recognizing and binding to specific receptors on target cells via their components and further activating intracellular signaling pathways. Given the multiple components in NETs, the receptors sensing NETs can also be diverse. Cell- or disease-specific receptors can exist under different conditions, even for the same component. Here, we initially discovered that cell surface ENO1 on CD4^+^ T cells acted as a receptor anchored by NET-MPO, facilitating NET accumulation around CD4^+^ T cells and recruiting IFITM2 as a DNA sensor in sepsis-induced immunosuppression.

ENO1 is a member of the enolase family and is functionally diverse in its localization. When expressed on the cell membrane, ENO1 functions as a plasminogen receptor, promoting the activation of plasminogen into plasmin (34). ENO1, expressed in the cytoplasm, acts as an essential rate-limiting enzyme for glycolysis, catalyzing the dehydration of 2-phosphoglyceric acid to phosphoenolpyruvate. ENO1 expression is associated with several diseases. However, the expression and function of ENO1 in sepsis still need to be clarified. In this study, we observed an increased expression of ENO1 in sepsis and found that it promoted the differentiation and function of Tregs. This is consistent with previous findings on the role of ENO1 in regulating immunosuppression under other conditions. For instance, nuclear ENO1 has been found to promote human Treg differentiation by modulating the expression of FOXP3 (35), while cell membrane ENO1 promotes pancreatic cancer cell proliferation (36). The mechanisms underlying the function of ENO1 are various. Here, we demonstrate a mechanism by which ENO1, the receptor of NET-MPO, encourages the accumulation of NETs around CD4^+^ T cells. However, the roles of ENO1 in the cytoplasm or nucleus in sepsis need to be further investigated.

Given its crucial role in pathogenesis, ENO1 is becoming a promising therapeutic target. It has been reported that knocking out *Eno1* significantly decreases tumor growth in mouse models (36) and that administering anti-ENO1 mAb inhibits the invasiveness of pancreatic cancer cells (37). In a septic mouse model, we observed decreased Treg differentiation and attenuated lethality following the secondary *P*. *aeruginosa* challenge upon chemical inhibition of ENO1. However, additional animal and cellular experiments should be conducted to further evaluate the therapeutic effects, and more specific and feasible ENO1 interventions should be explored.

IFITM2 is a broad-spectrum antiviral factor (38). Growing evidence suggests that IFITM2 plays a crucial role in regulating immune responses; for example, IFITM2 is upregulated during CD4^+^ T cell activation and regulates murine CD4^+^ Th cell differentiation (39). Therefore, the proinflammatory or antiinflammatory effects of IFITM2 largely depend on the specific immune cell types involved in inflammation in certain diseases. In this study, we identified the transmembrane protein IFITM2 as a DNA sensor that transmits the stimulatory signals of NETs and subsequently activates the cytoplasmic protein RAP1B and the ERK signaling pathway.

However, the specific activation antigens responsible for Treg induction during sepsis-induced immunosuppression remain to be fully characterized, representing a crucial area for future investigation. Several promising experimental approaches could be employed to address this knowledge gap, including single-cell RNA-Seq (40), high-resolution proteomic profiling (41), T cell receptor repertoire analysis (42), development of antigen-specific knockout animal models, and antigen activity screening platforms (43). Furthermore, the precise molecular mechanisms underlying ERK signaling pathway–mediated regulation of Treg differentiation and FOXP3 expression remain incompletely elucidated. A detailed understanding of these regulatory mechanisms will be a primary focus of our ongoing and future research efforts.

Although our in vitro studies have mechanistically demonstrated NET-MPO-ENO1–mediated Treg differentiation, we acknowledge that direct evidence of NETs regulating Tregs in sepsis-induced immunosuppression remains limited, which warrants further investigation through more sophisticated in vivo models. We recognize that static FOXP3 measurements cannot fully capture the spatiotemporal dynamics of Treg development. Implementing FOXP3 reporter mouse models in future investigations would enable precise tracking of Treg differentiation trajectories during sepsis progression, providing critical insights into the plasticity of these immunoregulatory cells. While the present study has principally elucidated NET-mediated pTreg regulation during the immunosuppression phase of sepsis, our observations of elevated NETs and expanded tTreg populations during the acute phase of sepsis suggest a potential role for NETs in tTreg biology, which is an intriguing possibility that requires dedicated investigation in future mechanistic studies.

Currently, the impact of gender on the prognosis of sepsis remains a matter of debate. Retrospective clinical analyses revealed that women might have worse outcomes for sepsis than men (44, 45). In contrast, some studies reported a significantly increased survival in the female group (46, 47). In this study, the age and gender of the healthy control group matched those of patients in the acute and immunosuppressive phases of sepsis. One limitation of this study is that we only used male mice for CLP modeling. However, some studies have indicated no significant immunopathological differences between the sexes in CLP-induced sepsis (48). We will further investigate the role of gender in the mechanisms by which NETs regulate Tregs in the future.

Overall, our study demonstrated the role and mechanism of NETs in regulating Treg differentiation and function during sepsis-induced immunosuppression, which may advance the understanding of the mechanism underlying the immunopathogenesis of sepsis. Moreover, the identified NET-MPO-ENO1-IFITM2 complex may serve as a potential therapeutic target for sepsis.

## Methods

### Sex as a biological variable.

Our study examined male mice because they exhibit less variability in phenotype.

### Patients.

According to the international guidelines for sepsis and septic shock (49) and the consensus on sepsis-induced immunosuppression (50), 16 patients diagnosed with sepsis for 1 or 14 days and admitted to the intensive care unit were included in this study. The healthy control group included 16 patients admitted to the hospital with no evidence of pulmonary infection and evaluated for solitary pulmonary nodules. The study collected demographic and clinical data, including sex, age, BMI, final diagnosis, comorbidities, sequential organ failure assessment, acute physiology and chronic health status scoring system II, and other information. Peripheral blood was harvested within 24 hours of admission to the hospital. The basic characteristics of the subjects included in this study are listed in Supplemental Table 1.

### Mice.

Male C57BL/6J mice (8–10 weeks old, 20–25 g of body weight), *Pad4^–/–^* transgenic mice, *Eno1^fl/fl^* transgenic mice, and *Cd4^Cre^* mice were obtained from the Shanghai Laboratory Animal Research Center (for further information, please refer to Supplemental Methods). The breeding involved crossing *Eno1^fl/fl^* transgenic mice with *Cd4^Cre^* mice to produce CD4^+^ T cell–specific Eno1-knockout mice (*Eno1^fl/fl^ Cd4^Cre^* mice). Validation of the *Eno1^fl/fl^ Cd4^Cre^* mice and genotyping primers are shown in Supplemental Figure 11 and Supplemental Table 5, respectively. All animals were maintained by the Department of Laboratory Animal Science of Fudan University under a 12-hour/12-hour light/dark cycle under specific pathogen-free conditions.

### Cell lines.

The human embryonic kidney cell line HEK293T was purchased from ATCC. Cells were identified by short tandem repeat analysis and tested negative for mycoplasma contamination. Cells were cultured in DMEM containing 10% FBS in a humidified incubator at 37°C with a 5% CO_2_ atmosphere.

### Sepsis model.

Mice were subjected to CLP to induce sepsis, as described previously (14). Briefly, after anesthesia, a 1 to 2 cm long abdominal incision was made, and the exposed cecum was then ligated from the distal cecum to the median of the ileocecal valve. A 22-gauge needle was used to penetrate once from the mesentery to the transmesentery, a small amount of feces was squeezed, and the muscle layer and skin layer were sutured sequentially after the cecum was recovered. Mice in the sham group received the same protocol without CLP. All mice received 0.9% saline and analgesia after the end of surgery and had free access to water and food. Surviving mice exhibit sustained immunosuppression after 8 days of CLP (51). Therefore, 1 week after CLP, the main observation time point for sepsis-induced immunosuppression was used.

### Additional methodological details.

For further information on neutrophil isolation and purification of NETs, naive CD4^+^ T cell isolation, generation of induced Tregs in vitro, nanoLC-MS/MS analysis, RNA-Seq, and other materials and methods, please refer to Supplemental Methods and Supplemental Tables 2–4.

### Statistics.

Data for this study are presented as mean ± SD, and all the statistical analyses were performed using SPSS 20.0 and GraphPad Prism 9.3 software. All experiments were independently replicated at least 3 times. The Shapiro-Wilk test was used to evaluate the normality of data. Two-tailed Student’s *t* test was used to compare the means between 2 independent samples. One-way ANOVA was used to compare the means of the independent samples among multiple groups, and 2-way ANOVA was performed to analyze time and intervention factors using the Tukey’s honestly significant difference test. Nonparametric data were assessed using the Mann-Whitney test. Survival was analyzed using the Kaplan-Meier method and compared with the log-rank test. A *P* value less than 0.05 was considered significant.

### Study approval.

This study was approved by the Ethics Committee of Zhongshan Hospital, Fudan University (B2021-182R) in accordance with the principles of the Declaration of Helsinki. Written informed consent was obtained from the patients or their relatives for this study. All animal experiments were performed according to the guidelines of the Animal Review Committee of Zhongshan Hospital, Fudan University (protocol license number 2020-119).

### Data availability.

The RNA-Seq data are available in the National Omics Data Encyclopedia (NODE) database (https://www.biosino.org/node/project/detail/OEP00005132). The nanoLC-MS/MS data have been uploaded to the NODE database (https://www.biosino.org/node/project/detail/OEP00006492 and https://www.biosino.org/node/project/detail/OEP00006495). Additional data supporting the study are available in the Supporting Data Values file and can also be obtained from the corresponding authors upon reasonable request.

## Author contributions

WC, JW, and JZ designed the study. YJ, SG, XL, HS, XW, JG, ZC, HW, XZ, and TZ performed the experiments. YJ, SG, XL, WC, JW, and JZ analyzed the data. YJ, SG, XL, WC, JW, and JZ wrote the paper. WC, JW, JZ, RBA, YL, TRB, and CM revised the manuscript. All authors gave final approval of the version to be published.

## Funding support

- National Natural Science Foundation of China (82272192, 82074538, and 82001550).
- National Natural Science Foundation of China (82572465, to WC).
- Shanghai Academic Research Leader Program (22XD1420400).
- Shanghai “Yiyuan New Star” Outstanding Youth Medical Talent Program (20244Z0012).
- Shanghai Municipal 2021 “Science and Technology Innovation Action Plan” (21S31902600).
- Innovative Research Team of High-Level Local Universities in Shanghai, Shanghai Key Laboratory for Acupuncture Mechanism and Acupoint Function (21DZ2271800).

## Supplementary Material

Supplemental data

Unedited blot and gel images

Supporting data values

## Version 1 09/02/2025

In-Press Preview

## Version 2 11/03/2025

Electronic publication

## Version 3 05/01/2026

Information regarding the validation of the Eno1fl/fl Cd4Cre mice used in this study was omitted in the original publication. The authors removed this information during the revision when they reduced the word count in the main manuscript to comply with journal length limits in the main manuscript. This information is now provided in Supplemental Figure 11 and Supplemental Table 5. In addition, the authors have further clarified the source of the mice. Lastly, during the preparation of this manuscript, a text error was introduced during copyediting by JCI staff. Specifically, an NIH public access statement was incorrectly included in the Funding Support section; however, no NIH funding was used for this study.
