# Corrigendum to Myeloperoxidase-anchored ENO1 mediates neutrophil extracellular trap DNA to enhance Treg differentiation via IFITM2 during sepsis

**DOI:** 10.1172/JCI206849

**Published:** 2026-05-01

**Authors:** Yi Jiang, Shenjia Gao, Xiya Li, Hao Sun, Xinyi Wu, Jiahui Gu, Zhaoyuan Chen, Han Wu, Xiaoqiang Zhao, Tongtong Zhang, Ronen Ben-Ami, Yuan Le, Timothy R. Billiar, Changhong Miao, Jie Zhang, Jun Wang, Wankun Chen

Original citation: *J Clin Invest*. 2025;135(21):e183541. https://doi.org/10.1172/JCI183541

Citation for this corrigendum: *J Clin Invest*. 2026;136(9):e206849. https://doi.org/10.1172/JCI206849

Information regarding the validation of the *Eno1^fl/fl^ Cd4^Cre^* mice used in this study was omitted in the original publication. The authors removed this information during the revision when they reduced the word count in the main manuscript to comply with journal length limits in the main manuscript. This information is now provided in Supplemental Figure 11 and Supplemental Table 5. In addition, the authors have further clarified the source of the mice. Lastly, during the preparation of this manuscript, a text error was introduced during copyediting by JCI staff. Specifically, an NIH public access statement was incorrectly included in the Funding Support section; however, no NIH funding was used for this study. The HTML and PDF versions of the article have been updated online.

## Version 1 05/01/2026

Electronic publication

## Version 2 05/04/2026

Removed "the authors regret the errors"

