# Supplementary material for: Myeloperoxidase-anchored ENO1 mediates neutrophil extracellular trap DNA to enhance Treg differentiation via IFITM2 during sepsis
Source: J Clin Invest. 2025 Sep 2;135(21):e183541. doi: 10.1172/JCI183541 (PMC12578386; doi:10.1172/JCI183541)
Supplement: Supplemental data [file jci-135-183541-s008.pdf]

Supplemental Materials

Supplementary Figures

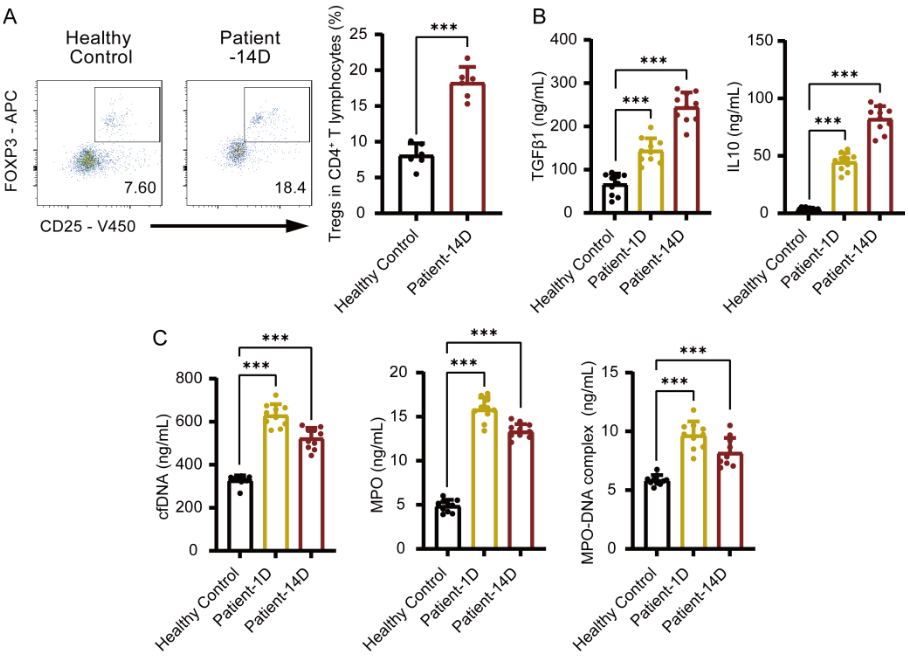

Figure S1. Tregs and NETs increased in human sepsis.

Peripheral blood was collected from patients with sepsis and healthy controls for further analysis. (A) The proportion of Tregs in the peripheral blood of healthy controls and patients in the immunosuppressive phase of sepsis was determined by flow cytometry (n=6). (B) The levels of TGFβ1 and IL10 were detected in the peripheral blood plasma of healthy controls and patients with sepsis (Patient-1D, Patient-14D) via ELISA (n=10). (C) CfDNA, MPO, and the MPO-DNA complex in the peripheral blood plasma of healthy controls and patients with sepsis (Patient-1D, Patient-14D) were quantified by the Quant-iT PicoGreen dsDNA Quantification Kit (cfDNA) and ELISA (MPO and the MPO-DNA complex) (n=10). Representative data are shown from 3 independent experiments (A-C). Data are represented as mean ± SD. ****P* < 0.001. Two-tailed student's t test was used for Figure A. One-way ANOVA was used for Figure B-C.

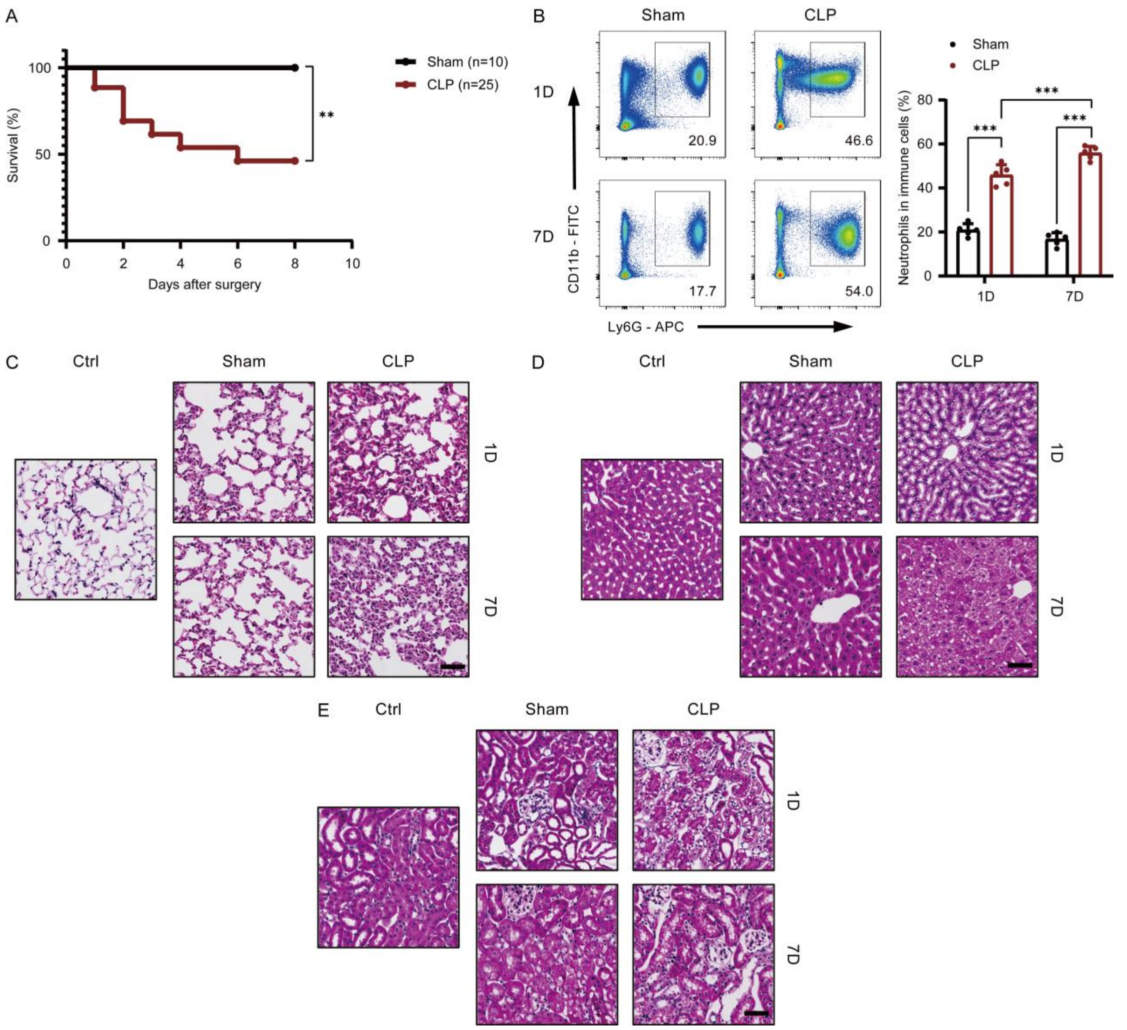

Figure S2. Sepsis was induced in mice using the cecal ligation and puncture (CLP) model. Mice were randomly subjected to CLP or sham operation to establish septic mice or sham mice. (A) Survival of CLP mice (n=25) and sham mice (n=10) was observed for 8 days and was shown by Kaplan-Meier survival curves. (B) The proportion of neutrophils in the peripheral blood of mice 1 day (1D) or 7 days (7D) after the CLP or sham procedure was analysed by flow cytometry (n=5). (C-E) H&E staining was conducted to assess lung (C), liver (D), and kidney (E) injury in CLP or sham mice at 1 day or 7 days post-operation. Mice that did not undergo any operation were used as a negative control (Ctrl). n=3 in each group. Scale bar = 50 μm. Representative data are shown from 3 independent experiments (A-E). Data are represented as mean ± SD. ***P* < 0.01, ****P* < 0.001. The Log-rank test was used for Figure A. Two-way ANOVA was used for Figure B.

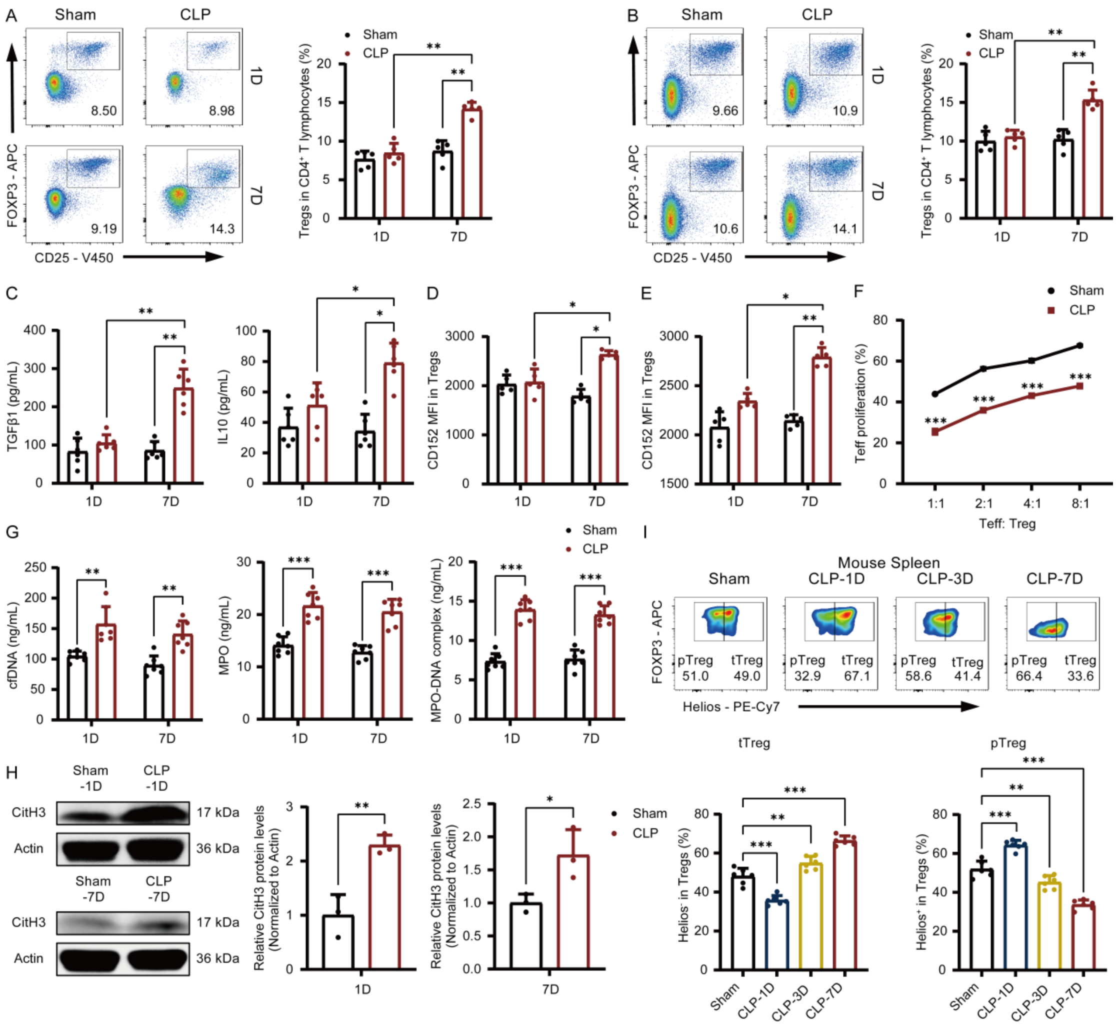

Figure S3. NETs release and Treg proportion were elevated in septic mice.

One day (1D) or 7 days (7D) after CLP or sham operation, peripheral blood and spleens were collected from each group of mice for subsequent experiments. (A) The proportion of Tregs in the peripheral blood of septic and sham mice was determined by flow cytometry (n=5). (B) The proportion of Tregs in the spleens of CLP and sham mice was detected by flow cytometry (n=5). (C) The levels of TGFβ1 and IL10 in the peripheral blood plasma of septic and sham mice were detected by ELISA (n=7). (D) The expression level of CTLA4 (CD152) in Tregs from the peripheral blood of septic and sham mice was determined by flow cytometry (n=5). (E) The expression level of CD152 in Tregs in the spleens of CLP and sham mice was measured by flow cytometry (n=5). (F) The suppressive capacity of splenic Tregs isolated from CLP-operated and sham mice at postoperative day 7 on Teff proliferation was evaluated (n=3). (G) cfDNA, MPO, and the MPO-DNA complex in the peripheral blood plasma of septic and sham mice were quantified by the Quant-iT PicoGreen dsDNA Quantification Kit (cfDNA) and ELISA (MPO and the MPO-DNA complex) (n=7). (H) CitH3 protein expression in the spleens of CLP and sham mice was measured by western blotting (n=3). (I) The proportion of tTregs and pTregs in the spleen of septic mice was determined by flow cytometry (n=6). Data are representative of 3 independent experiments (A-I). Data are represented as mean ± SD. **P* < 0.05, ***P* < 0.01, ****P* < 0.001. Two-way ANOVA was used for Figure A-G. Two-tailed student's t test was used for Figure H. One-way ANOVA was used for Figure I.

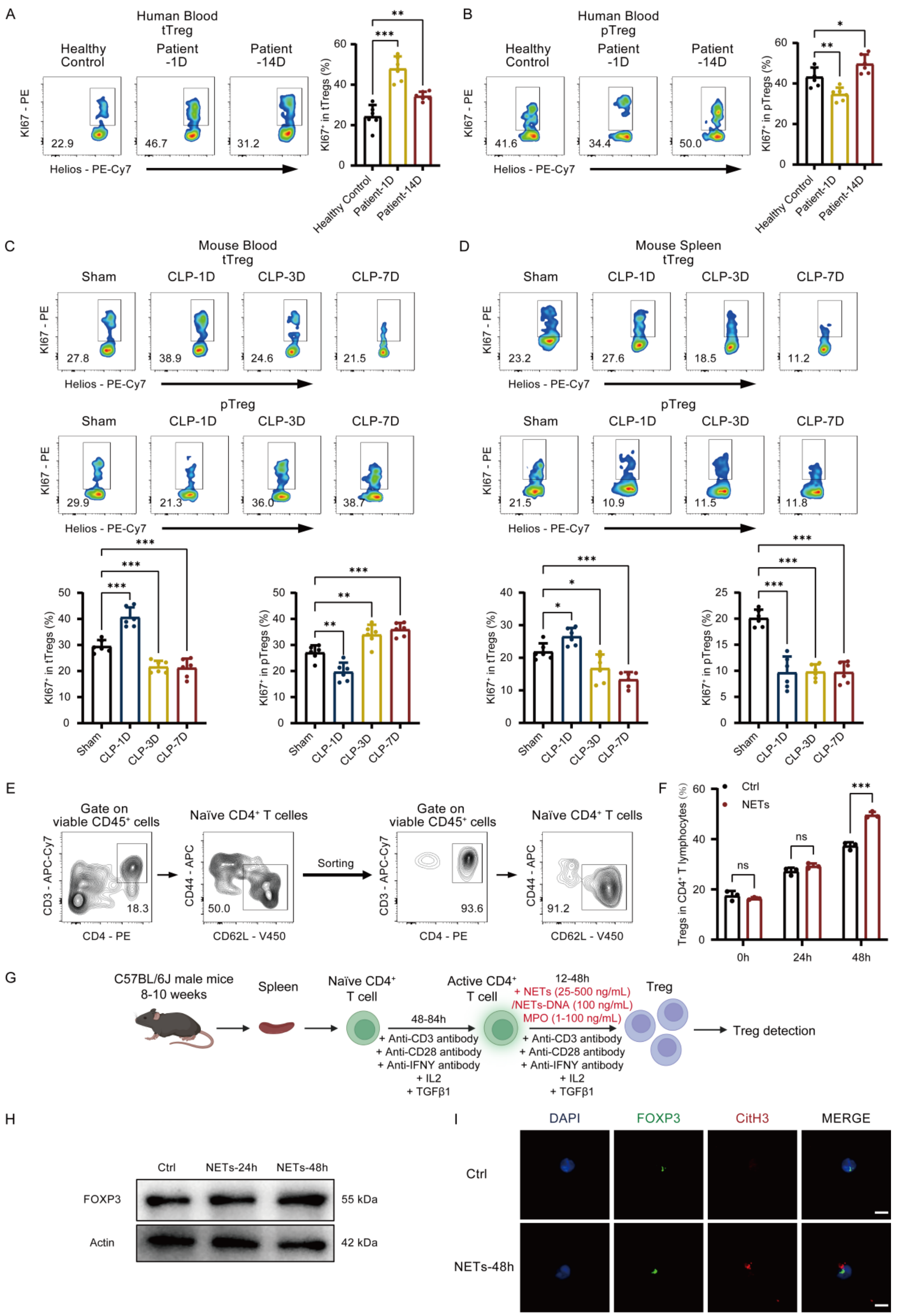

Figure S4. NETs promoted *in vitro* Treg differentiation.

(A-D) The proportion of KI67⁺ tTregs and KI67⁺ pTregs in the peripheral blood of septic patients and in the peripheral blood and spleen of septic mice was determined by flow cytometry (n=6). (E) Sorting and gating methods for isolating naïve CD4⁺ T cells (CD45⁺ CD4⁺ CD44^{lo} CD62L^{hi})

from murine spleens. (F) Naïve $CD4^+$ T cells were cultured under Treg-polarizing conditions and exposed to NETs (100 ng/mL) at various time points (0h, 24h, 48h) during the 48-hour differentiation period to assess their impact on Treg induction. Flow cytometry determined the proportion of induced Tregs (n=3). (G) Experimental protocol for inducing Tregs from naïve $CD4^+$ T cells and investigating the role of NETs during *in vitro* Treg differentiation. Sorted naïve $CD4^+$ T cells were seeded in anti-CD3-coated (10 μg/mL) 48-well plates and stimulated with anti-CD3 (2 μg/mL), anti-CD28 (2 μg/mL), anti-IFNγ (10 μg/mL), anti-IL4 (10 μg/mL), TGFβ1 (1.5 ng/mL) and IL2 (200 U/mL). NETs, purified NETs-DNA, and purified MPO were added to the medium after 48-84 hours. (H) Protein expression levels of FOXP3 in induced Tregs with (NETs-24h, NETs-48h) or without (Ctrl) NETs treatment (100 ng/mL) were measured by western blotting. (I) Immunofluorescence staining was conducted to observe the colocalization of NETs (100 ng/mL) and induced Tregs. Scale bar = 10 μm. Green: FOXP3; red: CitH3. Data are representative of 3 independent experiments (A-D, H-I). Data are represented as mean ± SD. ns denotes not significant, $^{*}P < 0.05$, $^{**}P < 0.01$, $^{***}P < 0.001$.One-way ANOVA was used for Figure A-D. Two way ANOVA was used for Figure F.

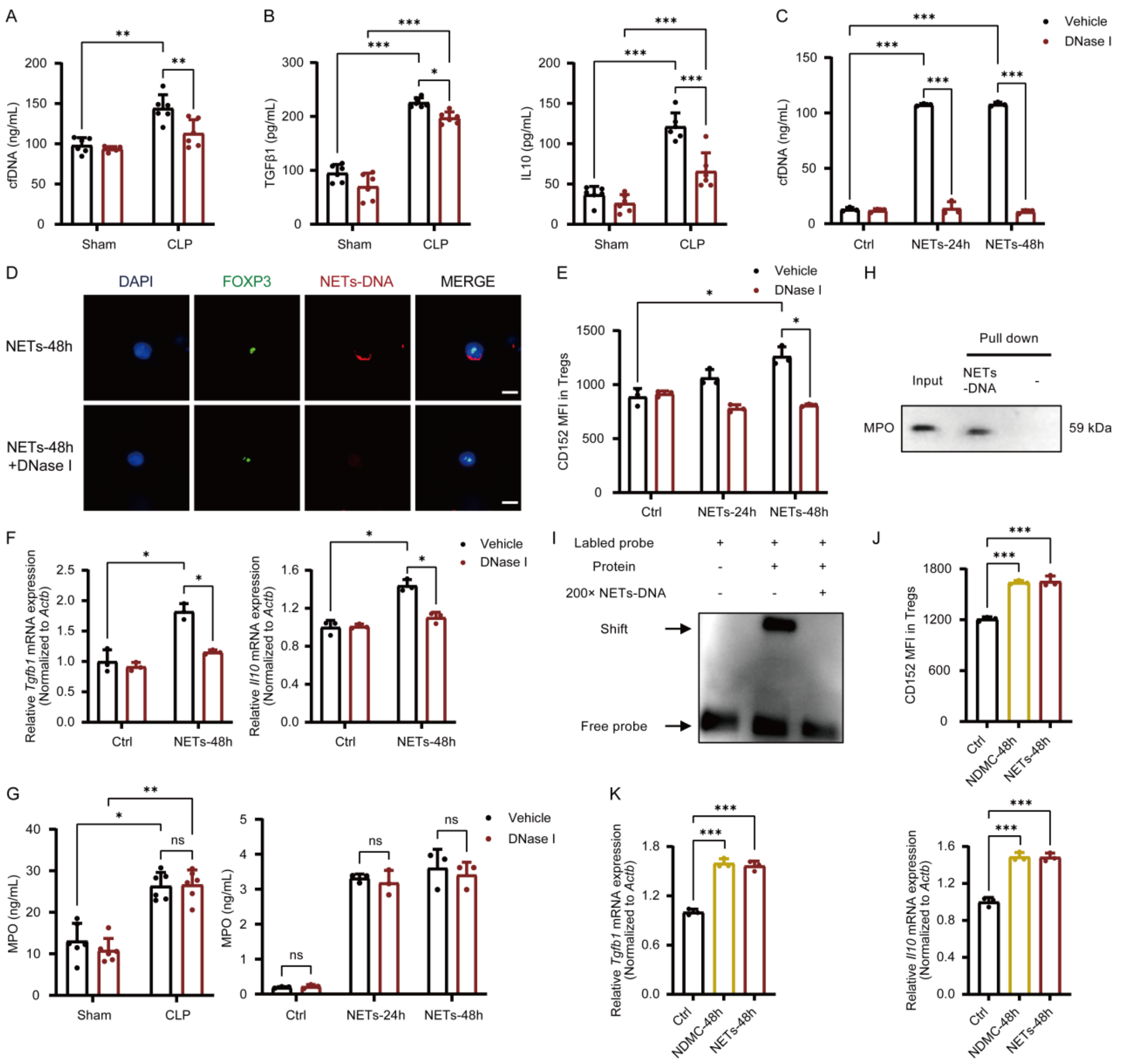

Figure S5. A complete structure is essential for NETs to facilitate Treg differentiation.

(A) Mice in the intervention group were given an *i.p.* injection of NETs-degrading DNase I (5 mg/kg), while vehicle-treated mice were given PBS daily from the day before CLP or sham operation until the end of the experiment. Peripheral blood plasma cfDNA was detected in different groups of mice (n=6). (B) The levels of TGFβ1 and IL10 in the peripheral blood plasma of CLP or sham mice treated with vehicle or DNase I (5 mg/kg) were quantified by ELISA (n=6). (C) *In vitro* Treg differentiation was induced with or without the treatment of NETs (100 ng/mL) or DNase I (2 μg/mL). The level of cfDNA in the culture media of *in vitro* Treg differentiation in different groups was assessed (n=3). (D) Immunofluorescence staining was conducted to observe the degradation of NETs-DNA and the colocalization of NETs (100 ng/mL) and induced Tregs. Scale bar = 5 μm. Green: FOXP3; red: NETs-DNA. (E) The expression of CD152 in Tregs obtained *in vitro* was detected by flow cytometry (n=3). (F) The mRNA expression of *Il10* and *Tgfb1* in Tregs differentiated *in vitro* was measured by qRT-PCR (n=3). (G) Left: the level of MPO in peripheral blood plasma in mice, *i.p.* administered DNase I (5 mg/kg) or vehicle, was determined by ELISA (n=6). Right: MPO levels in the culture media of *in vitro* Treg differentiation with or without NETs (100 ng/mL) or DNase I (2 μg/mL) were determined by ELISA (n=3). (H) Immunoblot analysis of purified MPO pulled down by biotinylated NETs-DNA (500 ng) and detected by MPO antibody. (I) Purified MPO (2 μg) was incubated with biotinylated NETs-DNA

(2 ng) with or without a 200-fold excess of unbiotinylated NETs-DNA. EMSA was performed to determine the binding of MPO to NETs-DNA. (J) NETs (100 ng/mL) or NDMC (100 ng/mL) were administered during *in vitro* Treg differentiation for 48 hours, and the expression of CD152 in induced Tregs was determined by flow cytometry (n=3). (K) The mRNA expression of *Il10* and *Tgfb1* in induced Tregs with or without NETs (100 ng/mL) or NDMC (100 ng/mL) for 48 hours was detected by qRT-PCR (n=3). Representative data are shown from 3 independent experiments (A-G, J-K). Data are represented as mean ± SD. ns denotes not significant, $*P < 0.05$, $**P < 0.01$, $***P < 0.001$. Two-way ANOVA was used for Figure A-C, E-G. One-way ANOVA was used for Figure J-K.

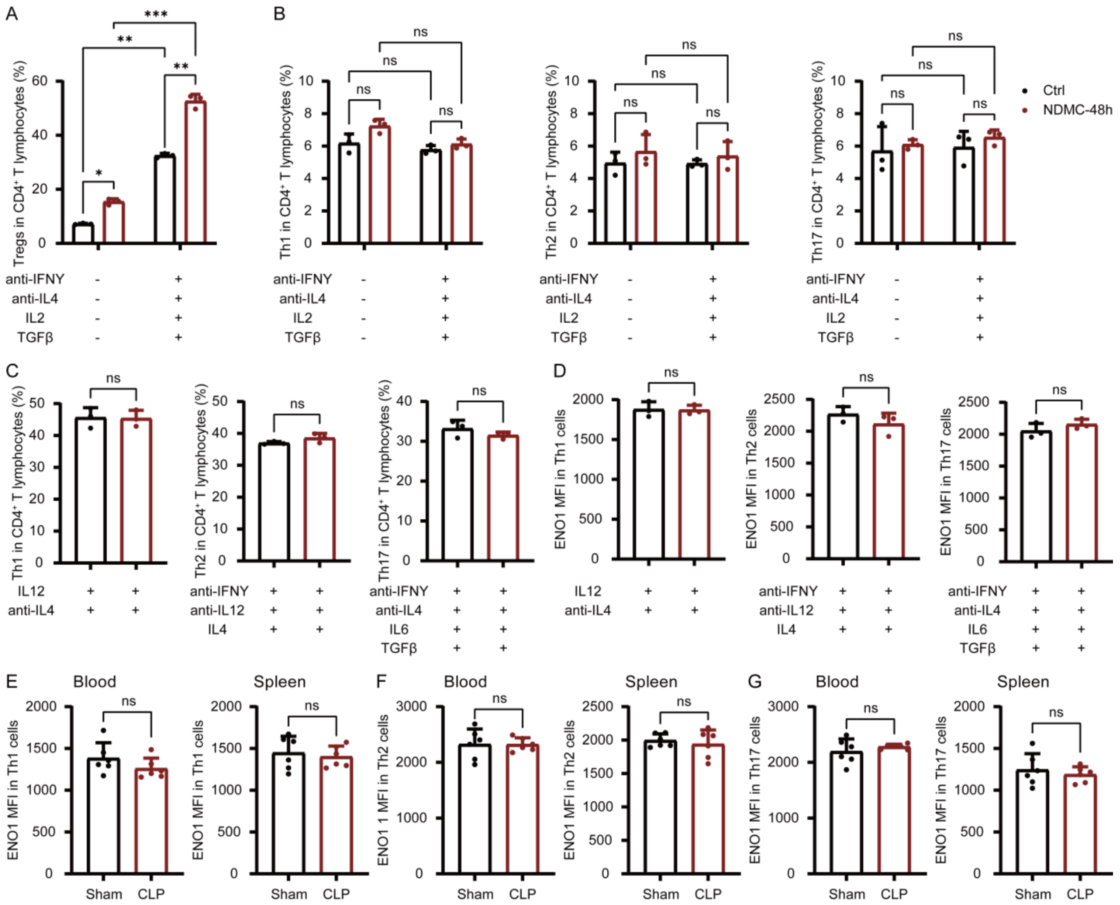

Figure S6. NETs exhibited no detectable effects on the differentiation of CD4⁺ T cell subsets other than Tregs.

(A-B) Naive CD4⁺ T cells were pre-stimulated for 48 hours, followed by NDMC treatment (100 ng/mL) for an additional 48 hours in media with or without Treg induction factors (anti-IFNγ (10 μg/mL), anti-IL4 (10 μg/mL), TGFβ1 (1.5 ng/mL) and IL2 (200 U/mL)). The proportions of induced Tregs, Th1, Th2, and Th17 cells were measured by flow cytometry (n=3). (C-D) For Th1 differentiation, naïve CD4⁺ T cells were stimulated with anti-CD3 (2 μg/mL) and anti-CD28 (2 μg/mL) in the presence of IL12 (10 ng/mL) and anti-IL4 (10 μg/mL) for 48 hours. Th2 polarization was induced using the same antibody stimulation combined with IL4 (20 ng/mL), anti-IFNγ (10 μg/mL), and anti-IL12 (5 μg/mL) for 48 hours. For Th17 differentiation, cells were activated under identical antibody conditions supplemented with TGFβ1 (2.5 ng/mL), IL6 (15 ng/mL), anti-IFNγ (10 μg/mL), and anti-IL4 (10 μg/mL) for 48 hours. NDMC (100 ng/mL) was administered during *in vitro* differentiation for additionally 48 hours. (C) The proportion of induced Th1, Th2, and Th17 cells was determined by flow cytometry (n=3). (D) The expression of ENO1 on induced Th1, Th2, and Th17 cells was evaluated by flow cytometry (n=3). (E-G) The expression of ENO1 on Th1, Th2, and Th17 cells in the spleens of CLP or sham mice 7 days post-operation was assessed by flow cytometry (n=6). Representative data are shown from 3 independent experiments (A-G). Data are represented as mean ± SD. ns denotes not significant, **P* < 0.05, ***P* < 0.01, ****P* < 0.001. Two-way ANOVA was used for Figure A-B. Two-tailed student's t test was used for Figure C-G.

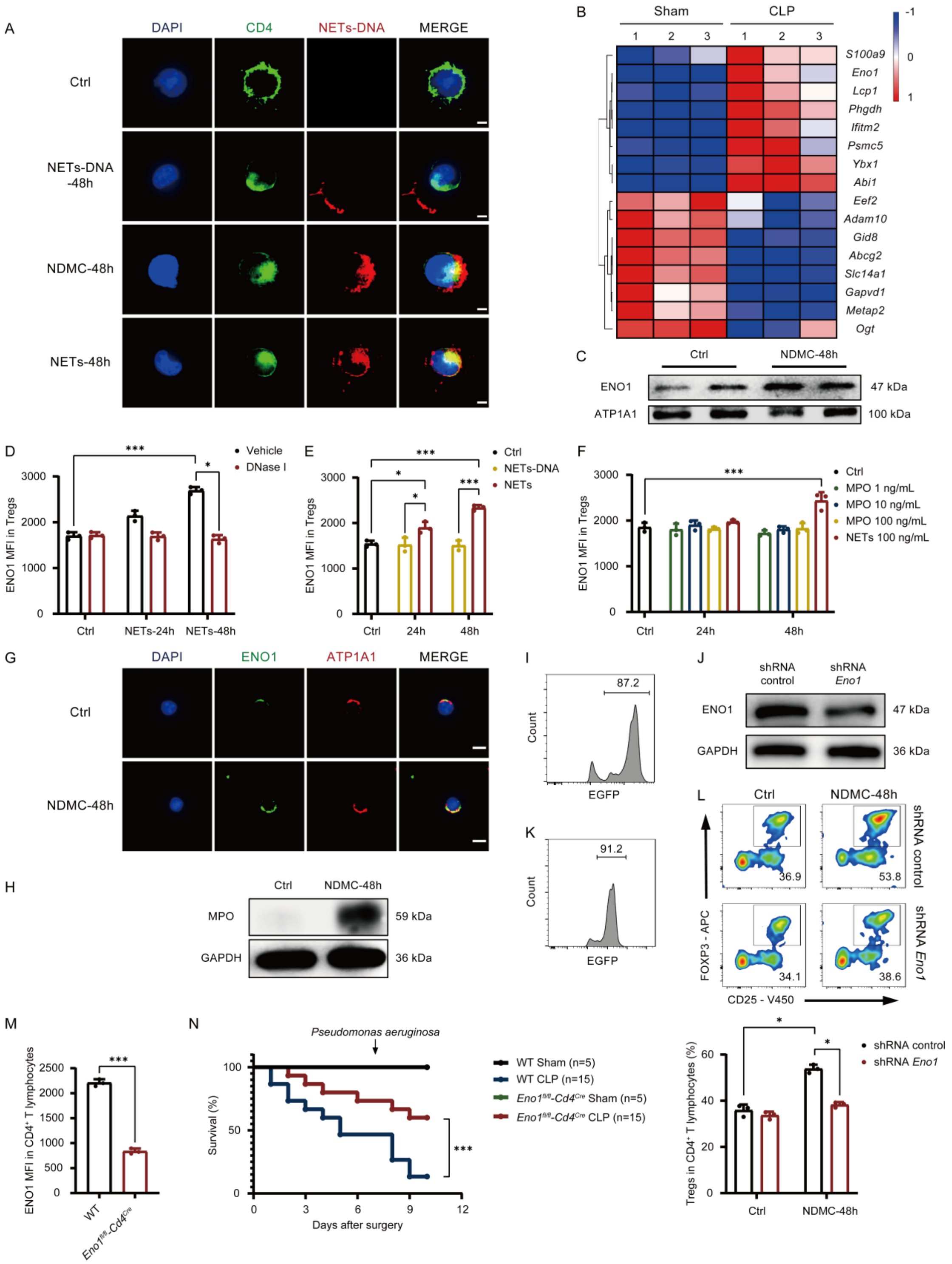

Figure S7. NETs-mediated Treg differentiation was dependent of ENO1.

(A) Immunofluorescence staining was conducted to observe the colocalization of NETs-DNA and $CD4^+$ T cells with or without treatments with NETs (100 ng/mL), the NETs-DNA-MPO complex (NDMC, 100ng/mL) or purified NETs-DNA (100 ng/mL). Scale bar = 5 μm. Green: CD4; red: NETs-DNA. (B) Total RNA was extracted from $CD4^+$ T cells isolated from the spleens of mice in the CLP and sham groups 7 days post operation, and RNA-seq was conducted. A heatmap of the differentially expressed genes encoding membrane proteins (CLP vs sham, *P* <0.05) was shown (n=3). (C) Naïve $CD4^+$ T cells under Treg differentiation conditions were

treated with NDMC (100 ng/mL) or vehicle, and the membrane expression of ENO1 was determined by western blotting. (D) The expression of ENO1 on Tregs induced *in vitro* with or without NDMC (100 ng/mL) or DNase I (2 μg/mL) treatment was detected by flow cytometry (n=3). (E) Tregs were induced *in vitro* in the presence of NDMC (100 ng/mL) or pure NETs-DNA (100 ng/mL). The expression of ENO1 on Tregs was detected by flow cytometry (n=3). (F) Tregs were induced *in vitro* in the presence of NDMC (100 ng/mL) or pure MPO (1, 10, 100 ng/mL). The expression of ENO1 on Tregs was measured by flow cytometry (n=3). (G) The colocalization of ENO1 and ATP1A1 (a protein used to indicate the cell membrane) on *in vitro*-induced Tregs treated with NDMC (100 ng/mL) or vehicle was observed by immunofluorescence staining. Scale bar = 5 μm. Green: ENO1; red: ATPA1. (H) Protein expression of MPO in naïve CD4$^+$ T cells under Treg differentiation conditions treated with NETs (500 ng/mL) or vehicle was determined by western blotting. (I-J) Naïve CD4$^+$ T cells were transfected with LV-shRNA control or LV-shRNA *Eno1* to knock down the *Eno1* gene and then induced into Tregs *in vitro*. Transduction efficiency was evaluated via flow cytometry 72 hours after lentiviral transduction of naïve CD4$^+$ T cells. (J) The protein expression of ENO1 was measured by western blotting. (K-L) Naïve CD4$^+$ T cells electroporated with LV-shRNA *Eno1* or LV-shRNA control plasmids were induced to differentiate into Tregs *in vitro* with or without NDMC treatment (100 ng/mL). (K) The transfection efficiency of T cell electroporation was assessed by flow cytometry at 24 hours post-transduction. (L) Flow cytometry evaluated the proportion of Tregs (n=3). (M) ENO1 expression in CD4$^+$ T cells in the spleens of wild-type (WT) mice or *Eno1*$^{fl/fl}$-*Cd4*^{Cre} mice was determined by flow cytometry (n=3). (N) The survival of WT mice or *Eno1*$^{fl/fl}$-*Cd4*^{Cre} mice that underwent CLP or sham procedure followed by nasal inhalation of *Pseudomonas aeruginosa* on the 7th day postoperation was observed for 10 days and was shown by Kaplan-Meier survival curves. WT sham (n=5), WT CLP (n=15), *Eno1*$^{fl/fl}$-*Cd4*^{Cre} sham (n=5), *Eno1*$^{fl/fl}$-*Cd4*^{Cre} CLP (n=15). Representative data are shown from 3 independent experiments (A, C-G, L, N). Data are represented as mean ± SD. **P* < 0.05, ****P* < 0.001. Two-way ANOVA was used for Figure D-F, L. Two-tailed student's t test was used for Figure M. The Log-rank test was used for Figure N.

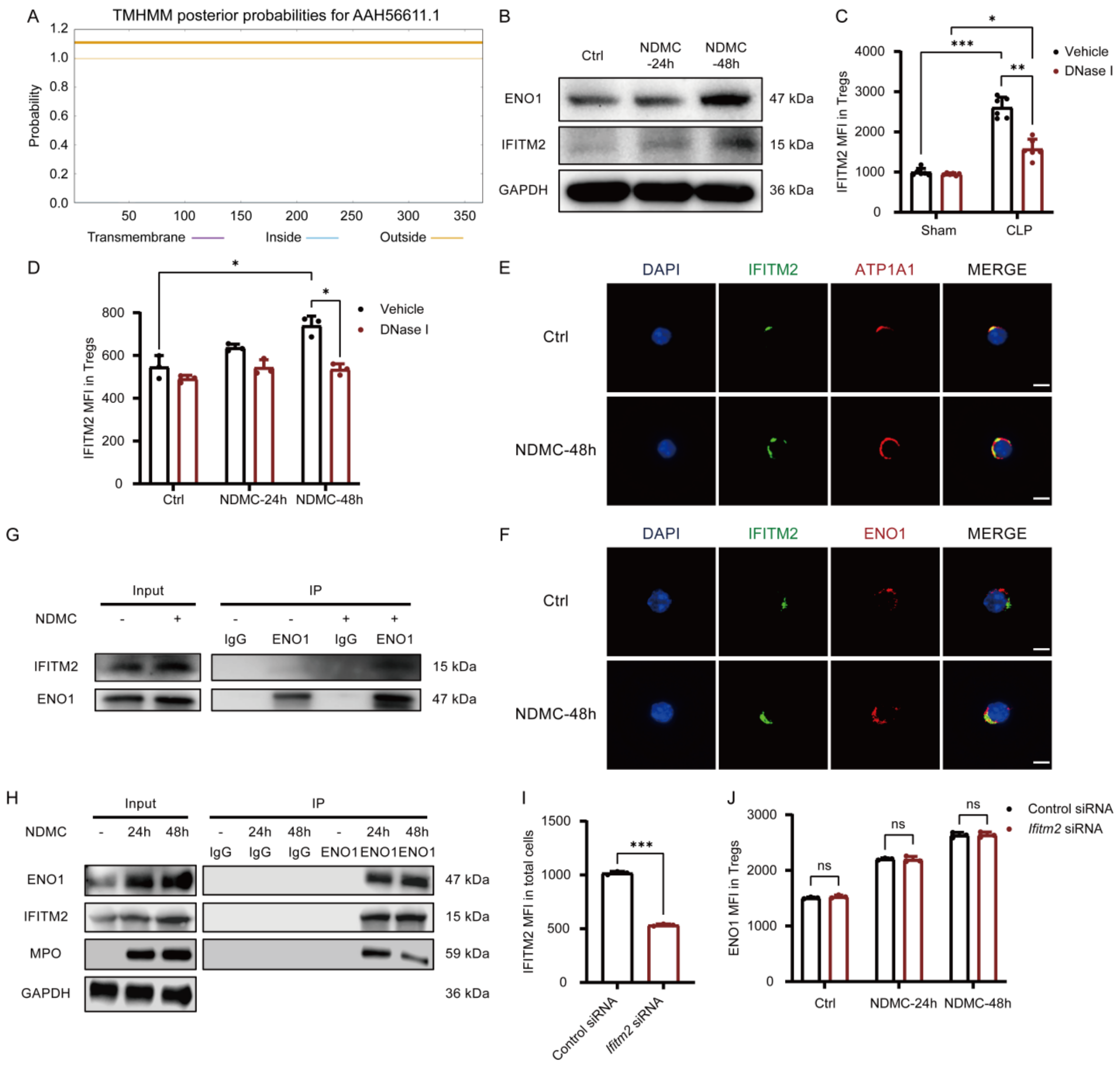

Figure S8. NETs regulated Tregs via MPO-ENO1-IFITM2 signal transmission.

(A) Transmembrane region prediction for ENO1 was conducted by TMHMM 2.0 (https://services.healthtech.dtu.dk/services/TMHMM-2.0/), and the transmembrane helix prediction result was presented. (B) The protein expression levels of IFITM2 and ENO1 in naïve CD4$^+$ T cells under Treg differentiation conditions treated with NDMC (100 ng/mL) or vehicle were determined by western blotting. (C) The expression of IFITM2 on Tregs in the spleens of CLP or sham mice treated with or without DNase I (5 mg/kg) was detected by flow cytometry (n=6). (D) The expression of IFITM2 on *in vitro*-induced Tregs treated with or without NDMC (100 ng/mL) or DNase I (2 μg/mL) was detected by flow cytometry (n=3). (E) The localization of IFITM2 was determined by immunofluorescence staining for IFITM2 and the membrane protein ATP1A1 in naïve CD4$^+$ T cells under Treg differentiation conditions treated with NDMC (100 ng/mL) or vehicle for 48 hours. Scale bar = 5 μm. Green: IFITM2; red: ATP1A1. (F) The colocalization of IFITM2 and ENO1 on naïve CD4$^+$ T cells under Treg differentiation conditions treated with NDMC (100 ng/mL) or vehicle was observed by immunofluorescence staining. Scale bar = 5 μm. Green: IFITM2; red: ENO1. (G) A pull down assay was conducted, and the immunoblot result for IFITM2 immunoprecipitated with ENO1 in naïve CD4$^+$ T cells under Treg differentiation conditions treated with NDMC (500 ng/mL) or vehicle was shown. (H) Immunoblot analysis of ENO1 and IFITM2 immunoprecipitated with MPO from naïve CD4$^+$ T

cells under Treg differentiation conditions treated with NDMC (500 ng/mL) or vehicle for 24 or 48 hours. (I) Naïve $CD4^+$ T cells were transfected with control siRNA or *Ifitm2* siRNA and then induced to differentiate into Tregs *in vitro* with or without the addition of NDMC (100 ng/mL). The expression of IFITM2 was measured by flow cytometry (n=3). (J) The expression of ENO1 on induced Tregs was determined by flow cytometry (n=3). Representative data are shown from 3 independent experiments (B-H, J). Data are represented as mean ± SD. ns denotes not significant, **P* < 0.05, ***P* < 0.01, ****P* < 0.001. Two-way ANOVA was used for Figure C-D, J. Two-tailed student's t test was used for Figure I.

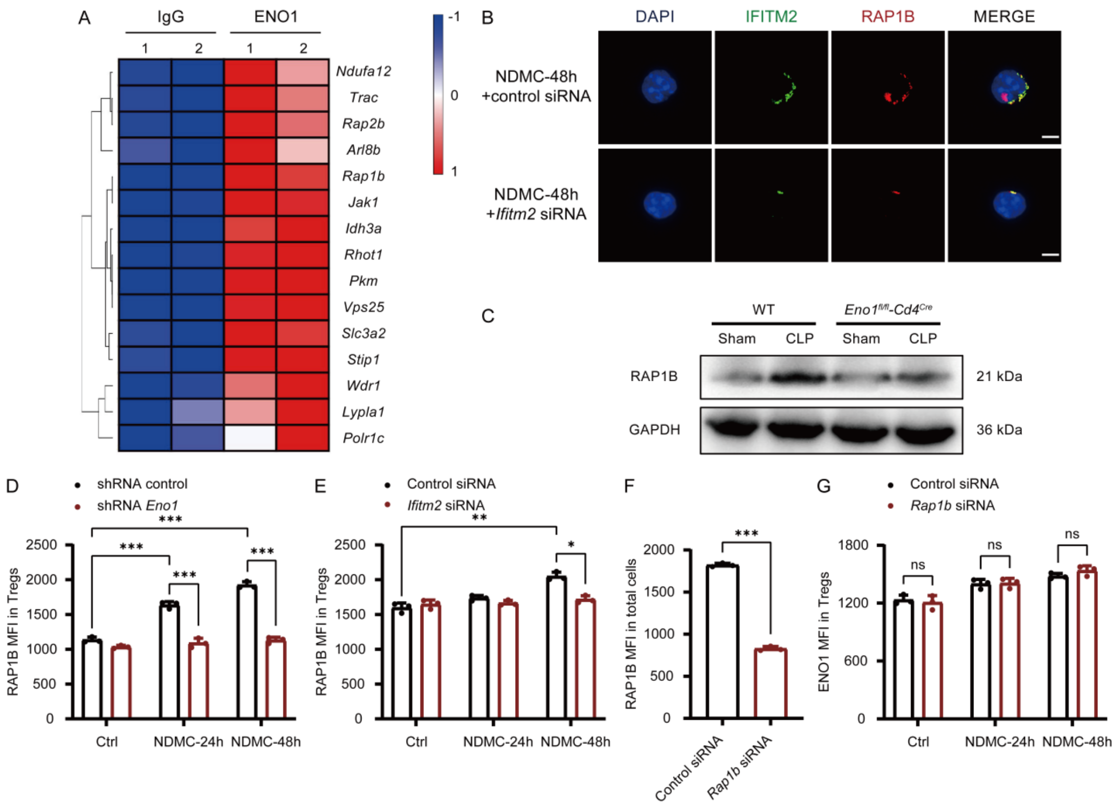

Figure S9. NETs-stimulation triggered the recruitment of RAP1 to ENO1-IFITM2.

(A) ENO1-IFITM2-binding cytoplasm proteins in naïve $CD4^+$ T cells under Treg differentiation conditions treated with NDMC (500 ng/mL) were shown in the heatmap (n=2). (B) Naïve $CD4^+$ T cells were transfected with control siRNA or *Ifitm2* siRNA and then induced to differentiate into Tregs after treatment with NDMC (100 ng/mL). The localization of RAP1B and IFITM2 was observed by immunofluorescence staining. Scale bar = 5 μm. Green: IFITM2; red: RAP1B. (C) RAP1B protein expression in Tregs from the spleens of WT mice or *Eno1*$^{fl/fl}$-*Cd4*^{Cre} mice 7 days after the CLP or sham procedure was evaluated by western blotting. (D) Naïve $CD4^+$ T cells were transfected with LV-shRNA control or LV-shRNA *Eno1* and then induced to differentiate into Tregs *in vitro* with or without NDMC (100 ng/mL). The expression of RAP1B in induced Tregs was determined by flow cytometry (n=3). (E) Naïve $CD4^+$ T cells were transfected with control siRNA or *Ifitm2* siRNA and then induced to differentiate into Tregs *in vitro* with or without NDMC (100 ng/mL). The expression of RAP1B in the obtained Tregs was assessed by flow cytometry (n=3). (F-G) Naïve $CD4^+$ T cells were transfected with control siRNA or *Rap1b* siRNA and then induced to differentiate into Tregs with or without NDMC treatment (100 ng/mL). (F) The expression of RAP1B was determined by flow cytometry (n=3). (G) The expression of ENO1 on induced Tregs was determined by flow cytometry (n=3). Data shown are representative of 3 independent experiments (B-E, G) and are presented as mean ± SD. ns denotes not significant, *$P < 0.05$, **$P < 0.01$, ***$P < 0.001$. Two-way ANOVA was used for Figure D-E, G. Two-tailed student's t test was used for Figure F.

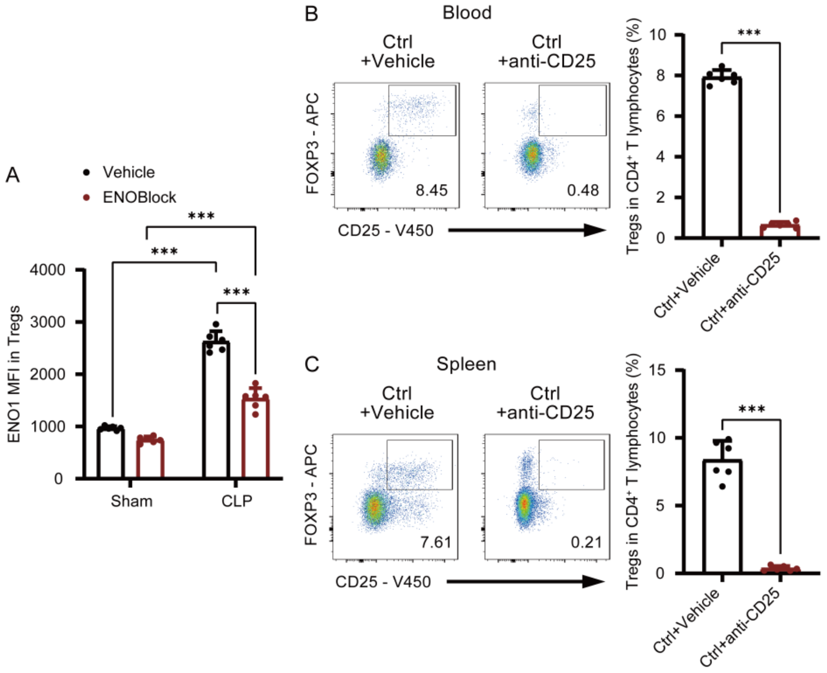

Figure S10. ENOBlock mediates protection during septic immunosuppression by suppressing Treg.

To assess the impact of ENO1 inhibition on sepsis-induced immunosuppression, mice received intraperitoneal injections of ENOBlock (10 mg/kg) every other day beginning 3 days post-surgery until experimental endpoint. Vehicle-control mice were administered solvent on the same schedule. (A) The expression of ENO1 on Tregs in the spleens of mice in different groups 7 days after the CLP or sham procedure was detected by flow cytometry (n=6). (B-C) Treg depletion was performed using anti-CD25 mAb (200 μg per mouse, 24 hours pre-*Pseudomonas aeruginosa* challenge). Treg proportions in the peripheral blood and spleen were analyzed by flow cytometry 72 hours post-challenge (n=6). Data shown are representative of 3 independent experiments (A) and are presented as mean ± SD. ***P < 0.001. Two-way ANOVA was used for Figure A. Two-tailed student's t test was used for Figure B-C.

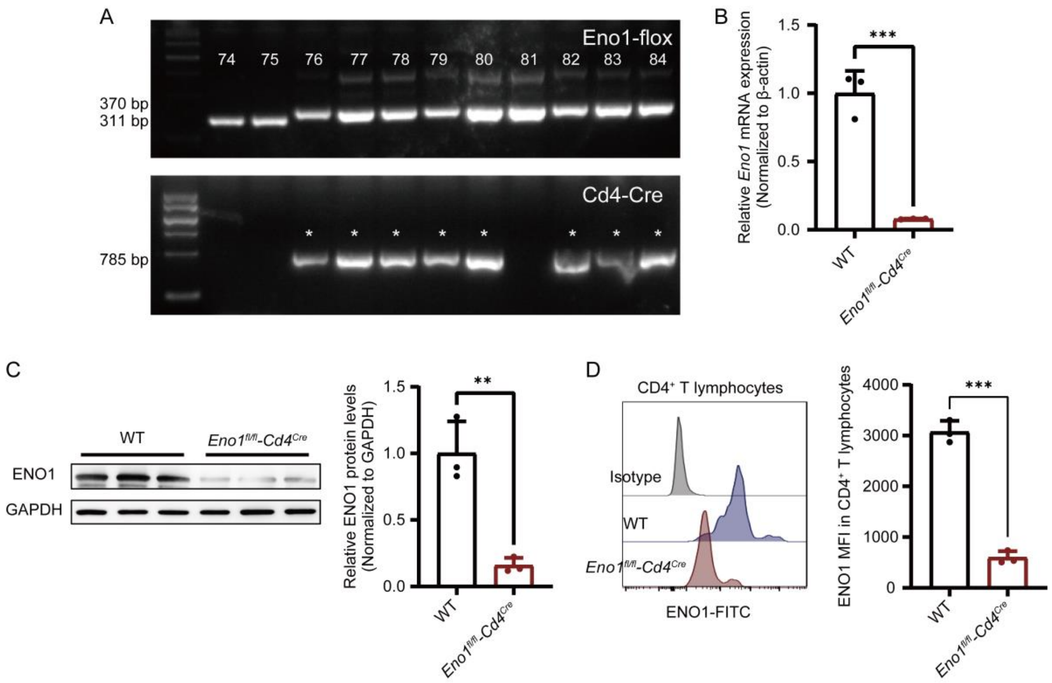

Figure S11. *Eno1$^{fl/fl}$-Cd4^{Cre}* mice used in this study were subjected to strain validation. (A) Mouse genotypes were determined by gel electrophoresis. For the *Eno1* flox allele, wild-type mice showed a single 311 bp band, heterozygotes showed 370 bp and 311 bp bands, and homozygotes showed a single 370 bp band. For the Cre transgene, mice with Cre activity were identified by a 785 bp band. (B) The mRNA expression of *Eno1* in CD4$^+$ T lymphocytes was measured by qRT-PCR (n=3). (C-D) ENO1 protein expression in CD4$^+$ T lymphocytes was measured by western blotting (C) and flow cytometry (D) (n=3). Data shown are representative of 3 independent experiments (A-D) and are presented as mean ± SD. $^{**}P < 0.01$, $^{***}P < 0.001$. Two-tailed student's t test was used for Figure B-D.

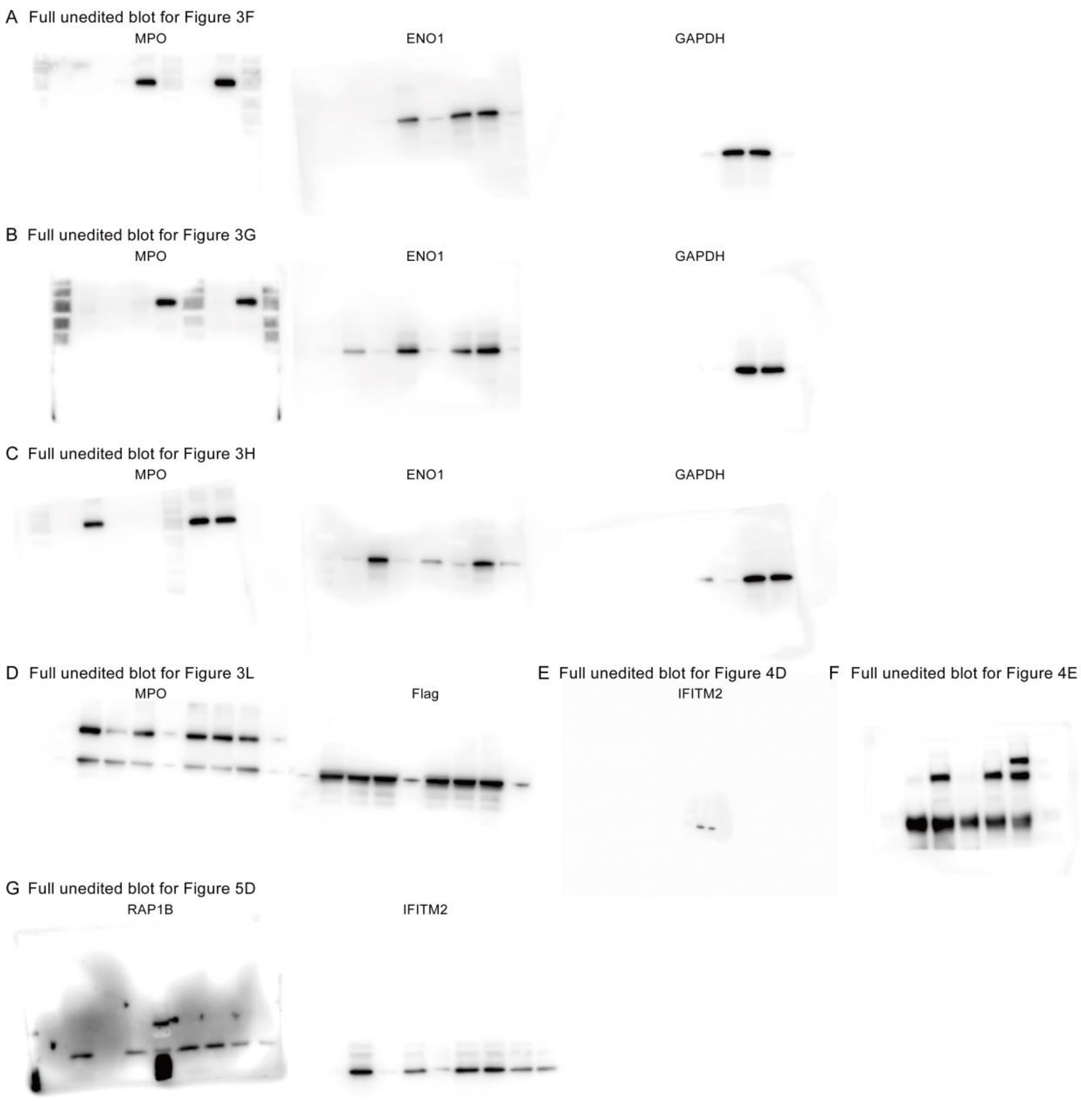
A Full unedited blot for Figure 3F
MPO
ENO1
GAPDH
B Full unedited blot for Figure 3G
MPO
ENO1
GAPDH
C Full unedited blot for Figure 3H
MPO
ENO1
GAPDH
D Full unedited blot for Figure 3L
MPO
Flag
E Full unedited blot for Figure 4D
IFITM2
F Full unedited blot for Figure 4E
G Full unedited blot for Figure 5D
RAP1B
IFITM2

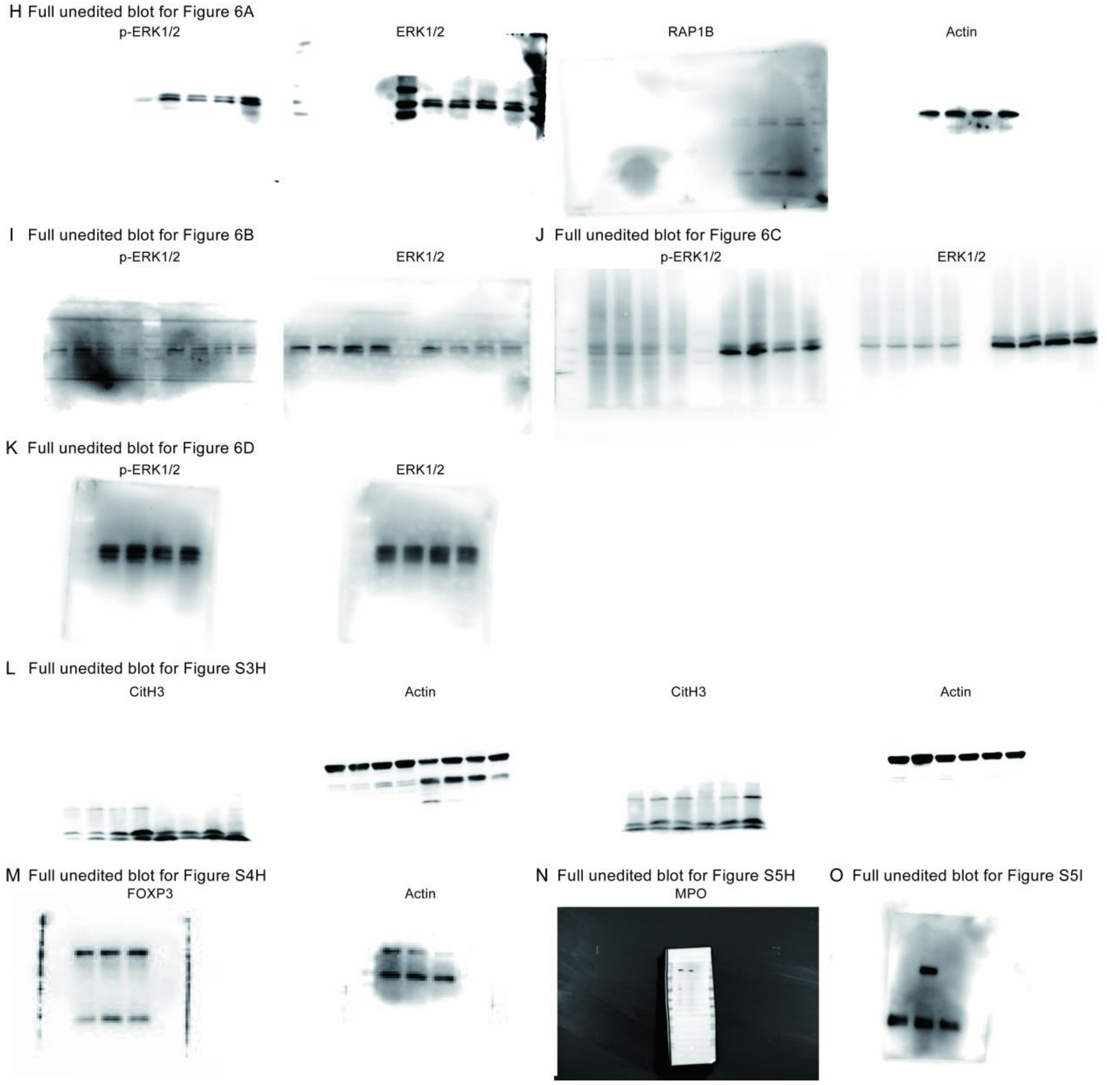
H Full unedited blot for Figure 6A
p-ERK1/2
ERK1/2
RAP1B
Actin
I Full unedited blot for Figure 6B
p-ERK1/2
ERK1/2
J Full unedited blot for Figure 6C
p-ERK1/2
ERK1/2
K Full unedited blot for Figure 6D
p-ERK1/2
ERK1/2
L Full unedited blot for Figure S3H
CitH3
Actin
CitH3
Actin
M Full unedited blot for Figure S4H
FOXP3
Actin
N Full unedited blot for Figure S5H
MPO
O Full unedited blot for Figure S5I

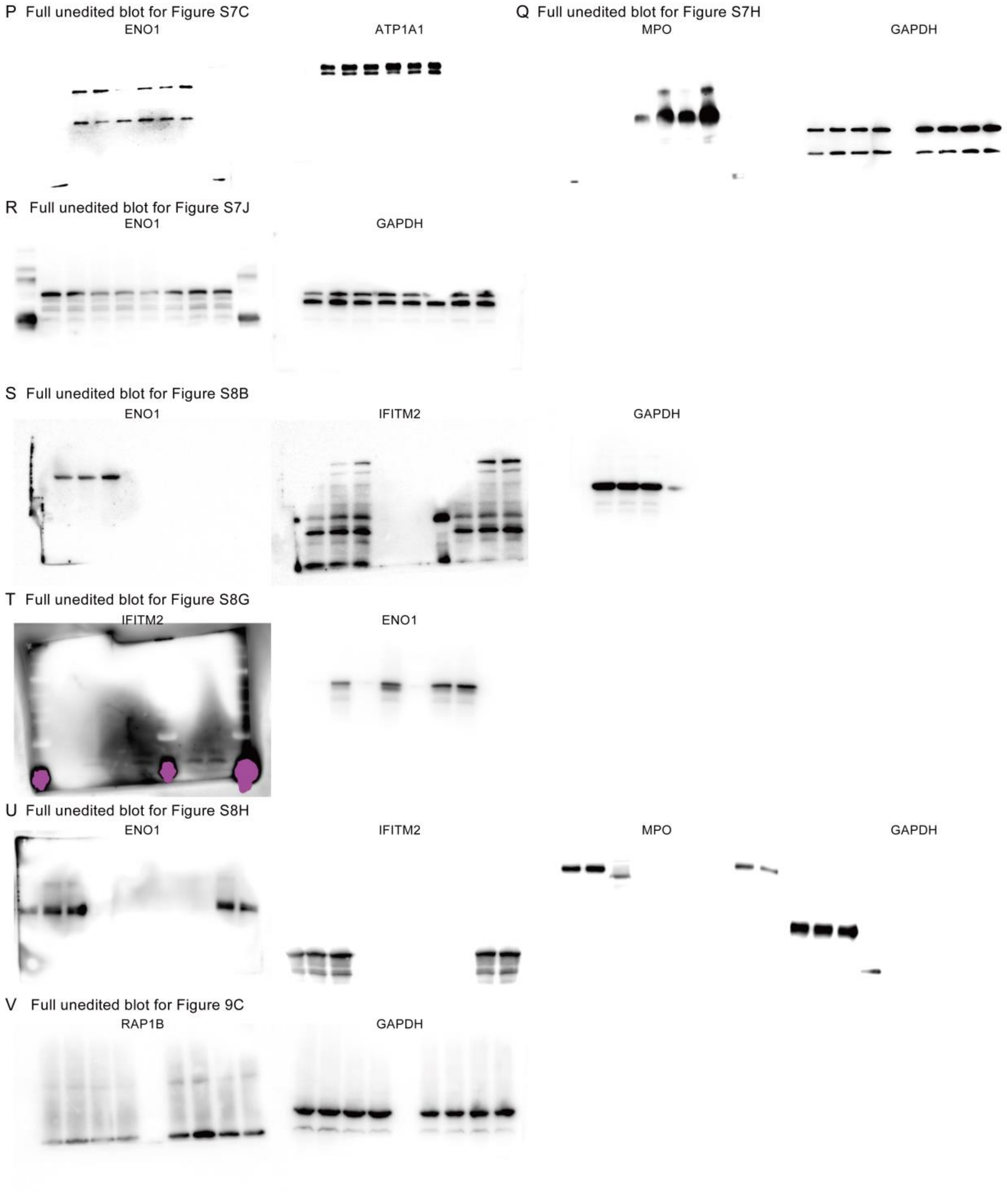

Figure S12. Full unedited blot.

Supplementary Tables

Table S1. Clinical characteristics of patients with sepsis and healthy control involved in this study.

	Sepsis patient-1D (n = 16)	Sepsis patient-14D (n = 16)	Healthy control (n = 16)
Age	58 (11)	61 (10)	60 (11)
Male sex	8 (50%)	8 (50%)	9 (56%)
BMI (kg/m^2)	23.4 (3.0)	16.5 (1.1)	24.0 (3.9)
ICU length of stay (d)	12 (6)	20 (6)	-
SOFA score	7.3 (2.2)	10.9 (4.1)	-
APACHEII	12.3 (3.6)	15.8 (3.9)	-
Mortality (14d)	4 (25%)	0 (0%)	-
Mortality (28d)	6 (38%)	5 (31%)	-
White cell count (10^9/L)	13.8 (2.8)	14.5 (2.8)	-
Lymphocyte count (10^9/L)	0.8 (0.17)	0.7 (0.14)	-
Serum albumin (mg/L)	35.4 (4.2)	25.4 (2.9)	-
CRP (μg/dL)	140.1 (18.6)	175.3 (12.3)	-

Note:

1. Data are n (%) or mean (SD) unless otherwise specified.
2. SOFA=sequential organ failure assessment on day of sampling.

Table S2. List of siRNA used in this study.

	Forward (5'-3')	Reverse (5'-3')
Ifitm2 siRNA-1	CAAGCUAUGAGACAAUCAAAGTT	CUUUGAUUGUCUCAUAGCUUGTT
Ifitm2 siRNA-2	CCUCUGUGGUAGUCUUUCAGUTT	ACUGAAAGACUACCACAGAGGTT
Ifitm2 siRNA-3	GCCUACUCUGUGAAGUCUAGGTT	CCUAGACUUCACAGAGUAGGCTT
Rap1b siRNA-1	GUGAAUAUAAGCUCGUCGUTT	ACGACGAGCUUAUAUUCACTT
Rap1b siRNA-2	CAGUCGACAUUUAACGACUTT	AGUCGUUAAAUGUCGACUGTT
Rap1b siRNA-3	CAACUGUGCAUUCUUAGAATT	UUCUAAGAAUGCACAGUUGTT
Control siRNA	UUCUCCGAACGUGUCACGUTT	ACGUGACACGUUCGGAGAATT

Table S3. List of primers used in quantitative real-time PCR.

	Forward (5’-3’)	Reverse (5’-3’)
Tgfb1	AACCAAGGAGACGGAATACA	CGTGGAGTTTGTTATCTTTGC
Il10	AGTGTGTATTGAGTCTGCTGG	GAGAGAGGTACAAACGAGGTT
Actb	CCTCTATGCCAACACAGT	AGCCACCAATCCACACAG

Table S4. List of shRNA used in this study.

	Forward (5'-3')	Reverse (5'-3')
Eno1 shRNA-1	CcggGGCACAGAGAATAAATCTAAACTCGAGTTTAGATTTATTCTCTGTGCCTTTTTTg	aattcaaaaaaGGCACAGAGAATAAATCTAAACTCGAGTTTAGATTTATTCTCTGTGCC
Eno1 shRNA-2	CcggCCCTAGAACTCCGAGACAATGCTCGAGCATTGTCTCGGAGTTCTAGGGTTTTTTg	aattcaaaaaaCCCTAGAACTCCGAGACAATGCTCGAGCATTGTCTCGGAGTTCTAGGG
Eno1 shRNA-3	CcggCGCATTGGAGCAGAGGTTTACCTCGAGGTAAACCTCTGCTCCAATGCGTTTTTTg	aattcaaaaaaCGCATTGGAGCAGAGGTTTACCTCGAGGTAAACCTCTGCTCCAATGCG
Control shRNA	CcggCCTAAGGTTAAGTCGCCCTCGCTCGAGCGAGGGCGACTTAACCTTAGGTTTTTTg	aattcaaaaaaCCTAAGGTTAAGTCGCCCTCGCTCGAGCGAGGGCGACTTAACCTTAGG

Table S5. List of primers used for genotyping analysis by gel electrophoresis.

	Forward (5'-3')	Reverse (5'-3')
Eno1 flox	CTCCCAGAAGGGTCTGATGC	CTGGCTCCTTAGCCCTGAGA
Cd4 Cre	AGGGTCGGAGACAATAACGG	TTGCGAACCTCATCACTCGT

Supplementary Methods

Mice

The transgenic mice used in this study were obtained from the Shanghai Laboratory Animal Research Center (SLARC). Due to our collaboration with SLARC, all transgenic animals used in this research were sourced from this center. The mice were originally provided to SLARC by the Shanghai Model Organisms Center (SMOC) under an official scientific research collaboration agreement.

Sepsis model

In the specific experiment, sham and septic mice were anesthetized with halothane and held vertically with the head facing upward. 40 μL of a bacterial suspension was slowly injected intranasally using an Eppendorf pipette to establish a second-hit model of sepsis (1). Survival was recorded three days after inhalation, and H&E staining was conducted to assess multiorgan injury. Bacteria were prepared as previously described (1, 2). *Pseudomonas aeruginosa* (ATCC, 27853) was grown overnight at 37 °C with constant shaking to the stationary phase in trypticase soy broth. The inoculum was prepared from overnight cultures grown on 5% blood agar plates (Servicebio, G3054). Serial dilutions and colony counts of either inocula corresponded to a density of ~1×10^9 colony-forming units (CFUs)/mL.

To investigate the effect of NETs on the progression of sepsis-induced immunosuppression, mice were given intraperitoneal injections of NETs-degrading DNase I (5 mg/kg, Roche, Cat # 11284932001) or the selective MPO inhibitor AZD5904 (10 mg/kg, Med Chem Express, Cat # HY-111341) daily from the day before surgery, and vehicle-treated mice were given PBS until the end of the experiment on 7 days after surgery (3-5). To evaluate the effect of ENO1 inhibition on the development of sepsis-induced immunosuppression, mice were *i.p.* injected with ENOBlock (10 mg/kg, Med Chem Express, Cat # 1177827-73-4), and sham mice were given solvent every other day from 3 days after surgery until the end of the experiment (6, 7). To determine the Treg dependence of ENOBlock's effects on septic survival, we performed Treg depletion via intraperitoneal injection of PC61 (anti-CD25 mAb) (200 μg/mouse; BioLegend, Cat #102002), administered 24 hours prior to the *Pseudomonas aeruginosa* challenge (8, 9). Mouse peripheral blood, spleen, lung, liver, and kidney were collected for flow cytometry, ELISA, western blotting, and histopathological analysis.

Quantification of cfDNA

Whole blood from humans and mice was harvested and stood at room temperature for 20 min. The peripheral blood plasma was collected and centrifuged at 3000 rpm for 20 min. The culture medium for Treg induction was collected after centrifugation at 3000 rpm for 20 min. The collected peripheral blood plasma and cell culture supernatants were immediately frozen in liquid nitrogen and stored at -80 °C for further analysis. Extracellular DNA was quantified using the Quant-iT PicoGreen dsDNA Quantification Kit (Invitrogen, Cat # P11496). According to the manufacturer's instructions, 10% plasma or cell culture supernatant was added to each well, and the plates were incubated for 5 min at room temperature in the dark. A multimode plate reader detected sample fluorescence (Ex=480 nm, Em=520 nm).

ELISA

The concentrations of MPO, the MPO-DNA complex, TGFβ1, and IL10 in the peripheral blood plasma and MPO and the MPO-DNA complex in the cell culture supernatant were determined using an ELISA kit (MULTISCIENCES, China) following the manufacturer's instructions.

Neutrophil isolation and purification of NETs

Bone marrow cells were collected from the femurs and tibias of mice, lysed with red blood cell lysis buffer (BioLegend, Cat # 420301), and resuspended in Hank's balanced salt solution. Purified mouse neutrophils were then obtained by centrifugation using Histopaque 1119 (Sigma-Aldrich, Cat # 11191) and Histopaque 1077 (Sigma-Aldrich, Cat # 10771) density gradients (10). The viability of the isolated neutrophils was > 93%, and the purity was > 90%. The obtained neutrophils (1×10^6/mL) were resuspended in RPMI 1640 medium supplemented with 10% fetal bovine serum (Gibco, Cat # 10099141C), 100 U/mL penicillin, and 100 mg/mL streptomycin.

The isolated neutrophils were stimulated with phorbol 12-myristate 13-acetate (PMA) (50 nM, Sigma-Aldrich, Cat # P1585) for 3.5 hours to induce the formation of NETs. Cells were then removed by centrifugation at 300 g for 5 min, followed by high-speed centrifugation at 18,000 g for 15 min to collect NETs (11). The protocol was established for obtaining neutrophil-formed NET complexes with DNA and all structural components (12-14).

NETs-DNA purification and biotinylation labeling

NETs-DNA was purified using the MicroElute DNA Purification Kit (OMEGA, Cat # D6296). Briefly, collected NETs were fragmented by sonication to a length of 200-500 bp (15), and then NETs-DNA was purified using a MicroElute HiBind DNA Column following the manufacturer's instructions. Following the quantification of cfDNA, 25 ng of purified MPO (Med Chem Express, Cat # HY-P70282) was incubated with 500 ng purified NETs-DNA overnight at 4 °C to prepare NETs-DNA-MPO complex (NDMC) (15).

The purified NETs-DNA was biotinylated using a Biotin 3' End DNA Labeling Kit (Thermo Scientific, Cat # 89818) according to the manufacturer's instructions. In brief, the sample labeling reactions were prepared by adding components in the order, substituting 5 pmol 3'-OH ends of the sample DNA in place of the Unlabeled Control Oligo, and adding a chloroform-isoamylol mixture to extract the TdT.

Naïve CD4⁺ T cell isolation and generation of induced Tregs in vitro

The spleens of 8- to 10-week-old wild-type (WT) mice were isolated, and single-cell suspensions were prepared. Naïve $CD4^+$ T cells were negatively sorted using the Mouse Naïve $CD4^+$ T Cell Isolation Kit (Miltenyi, Cat # 130-104-453) according to the manufacturer's instructions. The purity of the sorted naïve $CD4^+$ T cells was > 90%. Purified naïve $CD4^+$ T cells (2×10^5/mL) were resuspended in RPMI 1640 supplemented with 10% fetal bovine serum, 100 U/mL penicillin, and 100 mg/mL streptomycin, seeded in anti-CD3 (10 μg/mL, BioXCell, Cat # BE0001-1)-coated 48-well plates. For Treg differentiation, naïve $CD4^+$ T cells were stimulated with anti-CD3 (2 μg/mL), anti-CD28 (2 μg/mL, BioXCell, Cat # BE0015-1), anti-IFNγ (10 μg/mL, PeproTech, Cat # 500-P119), anti-IL4 (10 μg/mL, PeproTech, Cat # 500-P54), TGFβ1 (1.5

ng/mL, PeproTech, Cat # AF-100-21C), and IL2 (200 U/mL, PeproTech, Cat # 210-12) (16, 17). For Th1 differentiation, naïve $CD4^+$ T cells were stimulated with anti-CD3 (2 μg/mL), anti-CD28 (2 μg/mL), anti-IL4 (10 μg/mL), and IL12 (10 ng/mL, PeproTech, Cat # 212-12). For Th2 differentiation, naïve $CD4^+$ T cells were stimulated with anti-CD3 (2 μg/mL), anti-CD28 (2 μg/mL), anti-IFNγ (10 μg/mL), IL4 (20 ng/mL, PeproTech, Cat # 214-14) and anti-IL12 (5 mg/mL, BioXCell, Cat # BE0052). For Th17 differentiation, naïve $CD4^+$ T cells were stimulated with anti-CD3 (2 μg/mL), anti-CD28 (2 μg/mL), anti-IFNγ (10 μg/mL), anti-IL4 (10 μg/mL), TGFβ1 (2.5 ng/mL) and IL6 (15 ng/mL, PeproTech, Cat # 216-16) (16, 17).

After purification, seeding, and activation of naïve $CD4^+$ T cells for 48-84 hours, NETs (25 ng/mL, 50 ng/mL, 100 ng/mL, 500 ng/mL), NETs-DNA (100 ng/mL), MPO (1 ng/mL, 10 ng/mL, 100 ng/mL) and NETs-DNA-MPO complex (100 ng/mL) were added to the medium. Cells were then collected after 12-48 hours for analysis by flow cytometry, western blotting, and immunofluorescence to determine the generation of Tregs and to explore the role of NETs in Treg differentiation (11). For specific experimental purposes, DNase I (2 μg/mL) (11), the MPO inhibitor AZD5904 (10 μM) (18), or the ERK inhibitor SCH772984 (300 nM, Med Chem Express, Cat # HY-50846) (19) were added during the induction of Tregs. The differentiation and function of Tregs were then analyzed by flow cytometry.

***In vitro* Treg suppression assay**

To investigate the effects of NETs on the immunosuppressive function of Tregs, $CD4^+$ $CD25^+$ Tregs and $CD4^+$ $CD25^-$ effector T cells were isolated from the spleens of 8- to 10-week-old WT mice using the $CD4^+$ $CD25^+$ Regulatory T Cell Isolation Kit (Miltenyi, Cat # 130-091-041). According to the manufacturer's instructions, $CD4^+$ T cells were first negatively selected, followed by positive selection for $CD4^+$ $CD25^+$ Tregs using anti-PE beads. Unlabeled cells were designated as $CD4^+$ $CD25^-$ Teffs. Purified Tregs (2×10^5/mL) were cultured in anti-CD3 (10 μg/mL)-coated 48-well plates and activated by the addition of anti-CD3 (2 μg/mL), anti-CD28 (2 μg/mL), and IL2 (200 U/mL). Simultaneously, NETs (100 ng/mL), NDMC (100 ng/mL), DNase I (2 μg/mL), or PBS were added to the culture medium based on the experimental conditions. After 48 hours of stimulation, Tregs were centrifuged at 300 g for 5 min, and the supernatant was discarded. $CD4^+$ $CD25^-$ Teffs were labeled with 5 μM 5,6-carboxyfluorescein diacetate succinimidyl ester (CFSE) (BioLegend, Cat # 423801). The labeled Teffs were then mixed with pretreated Tregs at varying ratios (Teff: Treg = 1:1, 2:1, 4:1, 8:1) in a culture medium containing only anti-CD3 (2 μg/mL) and anti-CD28 (2 μg/mL). Cocultured cells were incubated at 37 °C with 5% CO_2 for 72 hours. The proliferation of Teffs was subsequently assessed by measuring CFSE dilution using flow cytometry (Wang H et al., 2021; Miyara M et al., 2009) (11, 20).

Vector constructs and transfection

We constructed the pcDNA3.1-Flag-ENO1 vector and various truncated mutants of *Eno1* from the full-length vector (Synbio Technology, China). According to the manufacturer's instructions, HEK293T cells were transfected with the above plasmids using Lipofectamine 2000 Transfection Reagent (Invitrogen, Cat # 11668019). Mouse *Eno1* shRNAs in the pSLenti-U6-shRNA-CMV-EGFP-F2A-Puro-WPRE vector were used for *Eno1* silencing (OBiO Technology, China). Lipofectamine 2000 Transfection Reagent was used to transfect siRNAs targeting *Ifitm2* and *Rap1b* and negative control siRNAs (OBiO Technology, China) into the *in*

vitro Treg induction system. The sequences of the sense strands of *Ifitm2* siRNA and *Rap1b* siRNA are listed in **Supplementary Table 2**.

Flow cytometry

In vitro-induced Tregs were collected, and single-cell suspensions were prepared from the peripheral blood of patients and mice, as well as from murine spleens. Cells were centrifuged and resuspended in cell staining buffer (BioLegend, Cat # 420201) and incubated with an anti-CD16/32 antibody (BioLegend, Cat # 101320) for 10 min at room temperature to block nonspecific binding. Fixable Viability Stain 510 (BD Biosciences, Cat # 564406), PerCP-Cy5.5-conjugated CD45 (BioLegend, Cat # 103132), FITC-conjugated CD11b (BioLegend, Cat # 101206), APC-conjugated LY6G (BioLegend, Cat # 127614), APC-Cy7-conjugated CD3 (BioLegend, Cat # 100222/317342), PE-conjugated CD4 (BioLegend, Cat # 100408), PerCP-Cy5.5-conjugated CD4 (BioLegend, Cat # 300530), PerCP-Cy5.5-conjugated CD8 (BioLegend, Cat # 100734), BV421-conjugated CD25 (BioLegend, Cat # 102034), PE-conjugated CD25 (BioLegend, Cat # 302606), BV421-conjugated CD127 (BioLegend, Cat # 351310), APC-conjugated CD44 (BioLegend, Cat # 103012), and BV421-conjugated CD62L (BioLegend, Cat # 104436) were incubated for the corresponding time. Cells were infiltrated with fixation/permeabilization buffer (BioLegend, Cat # 00-5523-00), followed by incubation with AF647-conjugated FOXP3 (BioLegend, Cat # 320014), PE-Cy7-conjugated CD152 (BioLegend, Cat # 106313), and PE-conjugated CD152 (BioLegend, Cat # 106305), PE-Cy7--conjugated Helios (BioLegend, Cat # 137236), PE-conjugated KI67 (BioLegend, Cat # 151210). For the experiments, anti-ENO1 (Proteintech, Cat # 11204-1-AP) or anti-IFITM2 (Proteintech, Cat # 12769-1-AP) antibodies were added before fixation/permeabilization and anti-RAP1B (Proteintech, Cat # 10840-1-AP) antibody was added after fixation/permeabilization to dilutions in an appropriate proportion, after which cells were resuspended in AF488-conjugated secondary antibody diluent (which was incubated at 4 °C, and the antibody dosage and incubation time were determined according to the product instructions). Stained cells were analyzed using FACSVerse™ flow cytometry, and data were analyzed using FlowJo 10.0.7 software.

Immunoprecipitation

In vitro induced Tregs treated for experimental purposes were collected and lysed in immunoprecipitation (IP) lysis buffer (Epizyme, Cat # PC105) supplemented with a protease inhibitor cocktail (Epizyme, Cat # GRF101) and a phosphatase inhibitor cocktail (Epizyme, Cat # GRF102). The lysates were incubated with antibodies against ENO1, MPO (Proteintech, Cat # 22225-1-AP), IFITM2, and the IgG control (Proteintech, Cat # 30000-0-AP) overnight at 4 °C, followed by incubation with Protein A/G Magnetic Beads (Epizyme, Cat # YJ003) overnight at 4 °C. The antigen-antibody-magnetic bead complex was washed 3 times using IP lysis buffer. The lysates of HEK293T cells transfected with plasmids were incubated with purified MPO (500 ng) for 1 hour at room temperature, followed by the addition of Anti-DYKDDDDK Magnetic Beads (Epizyme, Cat # YJ007) overnight at 4 °C, after which the protein-magnetic bead complex was washed three times with IP lysis buffer. The samples for SDS-PAGE detection were eluted with 1× SDS-PAGE loading buffer (Epizyme, Cat # LT101S) by boiling for 10 min. The samples employed for nanoflow liquid chromatography-tandem mass spectrometry

(NanoLC-MS/MS) analysis were eluted with elution buffer (Epizyme, Cat # YJ201) by incubating at room temperature for 10 min, and then 10 µL of neutralization buffer was added to every 100 µL of eluent, after which the pH of the elution product was adjusted to neutral.

Biotinylated NETs-DNA pull-down

Purified MPO or *in vitro*-induced Treg lysates were incubated with 500 ng biotinylated NETs-DNA in 500 µL IP lysis buffer overnight at 4 °C. The protein-DNA complex was then incubated with Streptavidin Magnetic Beads (Epizyme, Cat # YJ011) at room temperature for 1 hour. The beads were washed 3 times with IP lysis buffer and prepared for western blotting (21).

NanoLC-MS/MS analysis

Peptides were isolated using a NanoElute 2 ultra-performance liquid chromatography (Bruker, Germany) and then infused into a capillary source for ionization. Data were acquired by timsTOF Pro 2 mass spectrometry (Bruker, Germany). Separations were performed using PepSep C18 reversed-phase columns (1.9 µm, 75 µm×15 cm, Bruker, Germany). The mobile phases comprised solvent A (0.1% formic acid, 2% acetonitrile in water) and solvent B (0.1% formic acid in acetonitrile). Peptides were separated with a gradient of 0-9 min 6%-24% B, 9–11 min 24%-35% B, 11–13 min 35%-80% B, and 13–15 min 80% B at a constant flow rate of 500 nL/min. The applied electrospray voltage was 1.75 kV, and the precursors and fragments were analyzed with the TOF detector. The data-independent parallel accumulation serial fragmentation (dia-PASEF) mode was used for data acquisition. The full mass spectrometry scan was set at 300-1500 m/z, and 20 PASEF modes were acquired per cycle. The MS/MS scan range was 400-850 m/z, and the isolation window was set at 7 m/z.

Western blotting

Spleens were cut and weighed to an appropriate size, followed by adding RIPA lysis buffer (Epizyme, Cat # PC101) containing a protease inhibitor cocktail and a phosphatase inhibitor cocktail. Samples were ground with a tissue grinder and kept on ice for 15 min, and the supernatant was collected by centrifugation at 12000 rpm for 20 min. *In vitro*-induced Tregs were collected, and the supernatant was obtained after lysis and centrifugation. Cell membrane proteins were isolated using the Pierce Cell Surface Protein Isolation Kit (Thermo Scientific, Cat # 89881). Cells were labeled with EZ-Link Sulfo-NHS-SS-Biotin, and the biotinylated cell membrane proteins were purified with NeutrAvidin agarose and eluted.

The protein concentration was determined using the BCA Protein Detection Reagent (Thermo Fisher Scientific, Cat # 23225). Protein samples (30 µg) were separated by 10% SDS-PAGE and transferred to PVDF membranes (Millipore, Cat # ISEQ00010). PVDF membranes were blocked with 5% skim milk and incubated with antibodies against ENO1 (Proteintech, Cat # 67187-1-Ig), MPO (R&D Systems, Cat # AF3667), DYKDDDDK (Proteintech, Cat # 66008-4-Ig), IFITM2 (Proteintech, Cat # 66137-1-Ig), RAP1B, p-ERK1/2 (Cell Signaling Technology, Cat # 4370), ERK1/2 (Cell Signaling Technology, Cat # 4695), Cit-H3 (Abcam, Cat # ab5103), FOXP3 (Santa Cruz Biotechnology, Cat # sc-166212), Actin (Proteintech, Cat # 66009-1-Ig), GAPDH (Proteintech, Cat # 60004-1-Ig) and ATP1A1 (Proteintech, Cat # 14418-1-AP) at 4 °C overnight, followed by incubation with secondary antibodies (Absin, Cat #

abs20040/abs20039/abs20005) at room temperature for 1 hour. Bands were detected via an enhanced chemiluminescence (ECL) kit (Millipore, Cat # WBKLS0500) and an ImageQuant LAS 4000 imager. The gray value of each band was measured by ImageJ 1.8.0 software. The relative expression level of the target protein was calculated as the ratio of the gray value of the target protein to that of β-actin or GAPDH in the same sample. In contrast, the relative expression of p-ERK1/2 was the ratio of its gray value to that of ERK1/2 in the same sample.

EMSA

The EMSA assay followed the manufacturer's instructions using the LightShift Chemiluminescent EMSA Kit (Thermo Scientific, Cat # 20148). In brief, purified MPO (2 μg) or the membrane proteins of *in vitro*-induced Tregs (10 μg) were incubated with biotinylated NETs-DNA (1 ng) in the EMSA binding buffer with or without IFITM2 antibody (dilution ratio of 1:10), IgG control antibody (negative control) (dilution ratio of 1:10), or 200-fold excess of unbiotinylated NETs-DNA for 1 hour at room temperature. The samples were subjected to a 6% SDS-PAGE in 0.5 × Tris-borate-EDTA buffer for 1.5 hours at 100 V, and the proteins were transferred to a nylon membrane for 1 hour at 380 mA. The nylon membrane was then crosslinked at a distance of 1 cm from the membrane for 30 min with a UV lamp and incubated with stabilized streptavidin-horseradish peroxidase at room temperature for 1 hour. The biotin-labeled DNA was detected by chemiluminescence to determine the binding of MPO or IFITM2 to the DNA probe.

Immunofluorescence

In vitro-induced Tregs were fixed with 4% paraformaldehyde (PFA, Servicebio, Cat # G1101) at room temperature for 15 min and blocked with immunostaining blocking buffer (Beyotime, Cat # P0260) at room temperature for 1 hour. It was incubated at 4 °C overnight with antibodies against ENO1, MPO, IFITM2, RAP1B, ATP1A1 (Proteintech, Cat # 14418-1-AP), and CD4 (Proteintech, 67786-1-Ig). AF488-conjugated secondary antibodies (Invitrogen, Cat # A-11008/A-11001) or AF594-conjugated secondary antibodies (Invitrogen, Cat # A32740/A-11032/A32758) were then incubated for 1 hour at room temperature. Total and extracellular DNA was stained with 4',6-diamidino-2-phenylindole (DAPI) (SouthernBiotech, Cat # 0100-20) and SYTOX Orange Nucleic Acid Stains (Invitrogen, Cat # S11358) and visualized under an Olympus microscope.

H&E staining

The isolated lung, liver, and kidney tissues were washed with PBS, fixed with 4% PFA, dehydrated with an ethanol gradient, and paraffin-embedded to prepare 4 μm sections. Paraffin sections were deparaffinized, stained with hematoxylin and eosin, and finally visualized under light microscopy (Carl Zeiss, Jena, Germany).

qRT-PCR

Total RNA was extracted from *in vitro*-induced Tregs using TRIzol reagent (Invitrogen, Cat # 15596018), followed by reverse transcription into cDNA utilizing a PrimeScript RT Master Mix (TaKaRa, Cat # RR036A). Subsequently, real-time quantitative PCR was conducted with a TB Green Premix Ex Taq II Kit (TaKaRa, Cat # RR820A) according to the manufacturer's protocol.

Actb was used as the reference gene to assess the relative mRNA levels of *Tgfb1 and Il10*. The primer sequences for qRT-PCR are detailed in **Supplementary Table 3**.

T cell electroporation

To electroporate murine $CD4^+$ T cells, naïve $CD4^+$ T cells were isolated from mouse spleens and activated with anti-CD3 (2 μg/mL) and anti-CD28 (2 μg/mL) antibodies for 48 hours as described above. The cells were collected, washed twice in PBS, and resuspended at a concentration of 1×10^6/mL in Opti-MEM (Gibco, Cat # 31985070). 500 nM of siRNA or 20 μg of shRNA plasmids were added for each million cells. The cells were electroporated by Gene Pulser Xcell square wave electroporation system with a single 300 V pulse for 5 ms in 4 mm cuvettes at 4 °C. After electroporation, the samples were placed on ice and quickly transferred into a fresh culture medium and cultured at a density of 2×10^5 cells/mL (22). Transfection efficiency was assessed 48 hours later using flow cytometry or Western Blotting.

Lentiviral transduction of T cells

For stable transfection, $CD4^+$ T cells were infected with lentivirus (23, 24). The lentiviruses were packaged in HEK293T cells using GL427 and GL427NC2 plasmids. Murine $CD4^+$ T cells were activated with anti-CD3 (2 μg/mL) and anti-CD28 (2 μg/mL) antibodies for 48 hours and then infected with lentivirus in the presence of polybrene (10 μg/mL) (Sigma-Aldrich, Cat # H9268) for 24 hours. The infected $CD4^+$ T cells were centrifuged at 2000 g for 10 min at 37°C and incubated for 4 hours. Subsequently, the supernatant was replaced with fresh culture medium, and the cells were then cultured for an additional 48 hours. The shRNA sequences are listed in **Supplementary Table 4**.

RNA sequencing

Total RNA in $CD4^+$ T cells isolated from the spleens of mice in the CLP and sham groups 7 days post-operation was extracted using TRIzol reagent and detected on a 1% agarose gel. The purity, concentration, and integrity of the total RNA samples were assessed before further analysis. Ribosomal RNA from the samples was removed using an rRNA Depletion Kit (GenSeq, China) according to the manufacturer's instructions. Then, a sequencing library was constructed using a Low Input RNA Library Preparation Kit (GenSeq, China). The constructed sequencing libraries were qualitatively controlled and quantified on a BioAnalyzer 2100 System (Agilent Technologies, USA) and sequenced on an Illumina NovaSeq 6000 Sequencer to obtain raw data. Raw data quality control was performed using Cutadapt software to obtain high-quality and clean reads, which were then aligned to the reference genome using HISAT2 software. HTSeq software was then used to obtain the original counts, and the differentially expressed genes (DEGs) (P < 0.05) were ultimately screened and analyzed by the Kyoto Encyclopedia of Genes and Genomes (KEGG) pathway analysis.

References

1. Muenzer JT, et al. Pneumonia after cecal ligation and puncture: a clinically relevant "two-hit" model of sepsis. *Shock.* 2006;26(6):565-570.
2. Hotchkiss RS, et al. Role of apoptosis in Pseudomonas aeruginosa pneumonia. *Science.* 2001;294(5548):1783.
3. Aramburu IV, et al. Functional proteomic profiling links deficient DNA clearance with increased mortality in individuals with severe COVID-19 pneumonia. (1097-4180 (Electronic)).
4. Duan ZA-O, et al. De-escalation antibiotic therapy alleviates organ injury through modulation of NETs formation during sepsis. (2058-7716 (Print)).
5. Liu TW, et al. Inhibition of myeloperoxidase enhances immune checkpoint therapy for melanoma. *J Immunother Cancer.* 2023;11(2).
6. Luo Y, et al. Keratin 17 covalently binds to alpha-enolase and exacerbates proliferation of keratinocytes in psoriasis. (1449-2288 (Electronic)).
7. Shi YA-O, et al. Targeting Endothelial ENO1 (Alpha-Enolase) -PI3K-Akt-mTOR Axis Alleviates Hypoxic Pulmonary Hypertension. (1524-4563 (Electronic)).
8. Hu ZQ, et al. Partial Depletion of Regulatory T Cells Enhances Host Inflammatory Response Against Acute Pseudomonas aeruginosa Infection After Sepsis. *Inflammation.* 2018;41(5):1780-1790.
9. Zouggari Y, et al. Regulatory T cells modulate postischemic neovascularization. *Circulation.* 2009;120(14):1415-1425.
10. Jia J, et al. Ferritin triggers neutrophil extracellular trap-mediated cytokine storm through Msr1 contributing to adult-onset Still's disease pathogenesis. *Nat Commun.* 2022;13(1):6804.
11. Wang H, et al. Regulatory T-cell and neutrophil extracellular trap interaction contributes to carcinogenesis in non-alcoholic steatohepatitis. *J Hepatol.* 2021;75(6):1271-1283.
12. Yazdani HO, et al. Neutrophil Extracellular Traps Drive Mitochondrial Homeostasis in Tumors to Augment Growth. *Cancer Res.* 2019;79(21):5626-5639.
13. Yang LY, et al. Increased neutrophil extracellular traps promote metastasis potential of hepatocellular carcinoma via provoking tumorous inflammatory response. *J Hematol Oncol.* 2020;13(1):3.
14. Tohme S, et al. Neutrophil Extracellular Traps Promote the Development and Progression of Liver Metastases after Surgical Stress. *Cancer Res.* 2016;76(6):1367-1380.
15. Yang L, et al. DNA of neutrophil extracellular traps promotes cancer metastasis via CCDC25. *Nature.* 2020;583(7814):133-138.
16. Yu T, et al. TRIM11 attenuates Treg cell differentiation by p62-selective autophagic degradation of AIM2. *Cell Rep.* 2023;42(10):113231.
17. Carriche GM, et al. Regulating T-cell differentiation through the polyamine spermidine. *J Allergy Clin Immunol.* 2021;147(1):335-348.e311.
18. Ramachandra CJA, et al. Inhibiting cardiac myeloperoxidase alleviates the relaxation defect in hypertrophic cardiomyocytes. *Cardiovasc Res.* 2022;118(2):517-530.
19. Morris EJ, et al. Discovery of a novel ERK inhibitor with activity in models of acquired resistance to BRAF and MEK inhibitors. (2159-8290 (Electronic)).
20. Miyara M, et al. Functional delineation and differentiation dynamics of human CD4+ T cells expressing the FoxP3 transcription factor. *Immunity.* 2009;30(6):899-911.
21. Abe T, et al. STING recognition of cytoplasmic DNA instigates cellular defense. *Mol Cell.*

2013;50(1):5-15.

22. Tilsed CM, et al. IL7 increases targeted lipid nanoparticle-mediated mRNA expression in T cells in vitro and in vivo by enhancing T cell protein translation. *Proc Natl Acad Sci U S A.* 2024;121(13):e2319856121.

23. Yang Y, et al. Suppression of non-muscle myosin II boosts T cell cytotoxicity against tumors. *Sci Adv.* 2024;10(44):eadp0631.

24. Zeng S, et al. Local TSH/TSHR signaling promotes CD8(+) T cell exhaustion and immune evasion in colorectal carcinoma. *Cancer Commun (Lond).* 2024;44(11):1287-1310.
