# Supplementary material for: Myeloperoxidase-anchored ENO1 mediates neutrophil extracellular trap DNA to enhance Treg differentiation via IFITM2 during sepsis
Source: J Clin Invest. 2025 Sep 2;135(21):e183541. doi: 10.1172/JCI183541 (PMC12578386; doi:10.1172/JCI183541)

A Full unedited blot for Figure 3F

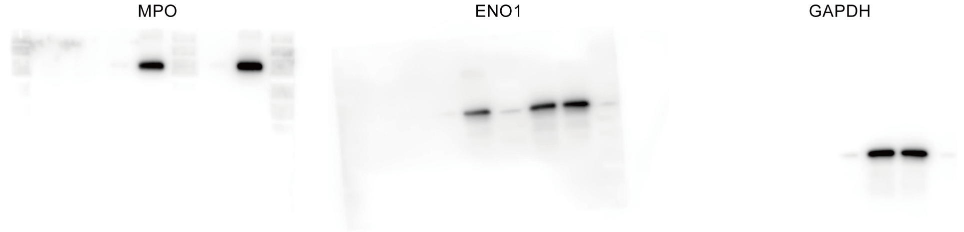

B Full unedited blot for Figure 3G

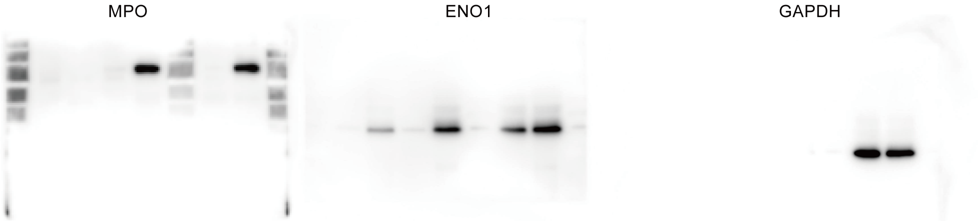

C Full unedited blot for Figure 3H

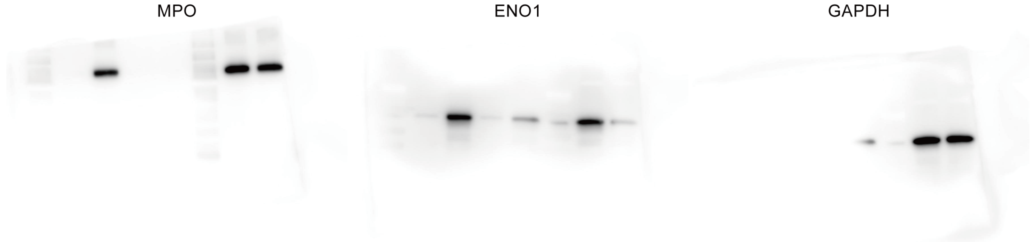

D Full unedited blot for Figure 3L

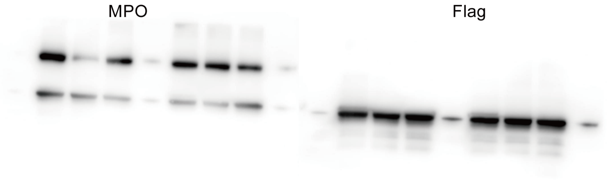

E Full unedited blot for Figure 4D

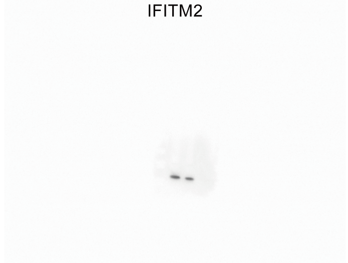

F Full unedited blot for Figure 4E

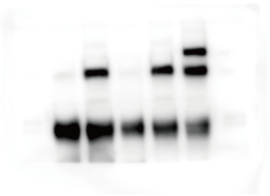

G Full unedited blot for Figure 5D

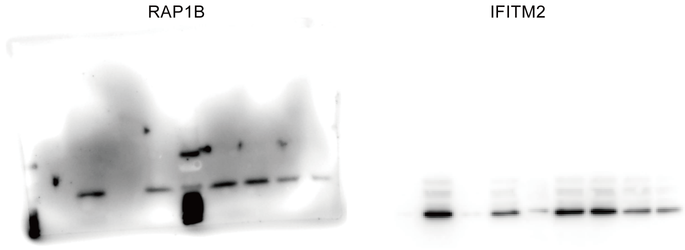

H Full unedited blot for Figure 6A

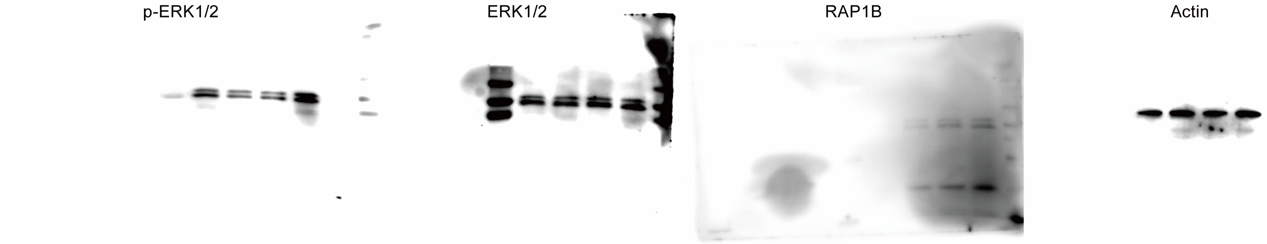

I Full unedited blot for Figure 6B

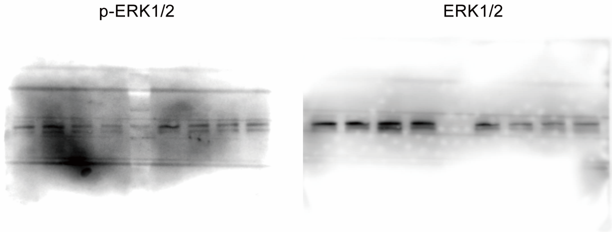

J Full unedited blot for Figure 6C

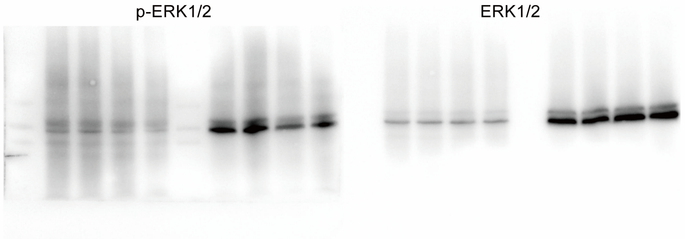

K Full unedited blot for Figure 6D

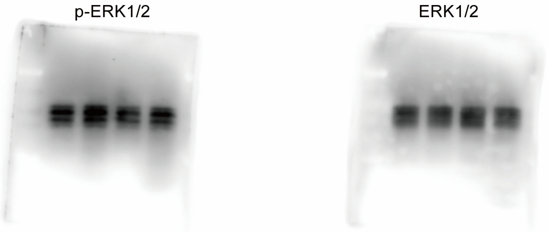

L Full unedited blot for Figure S3H

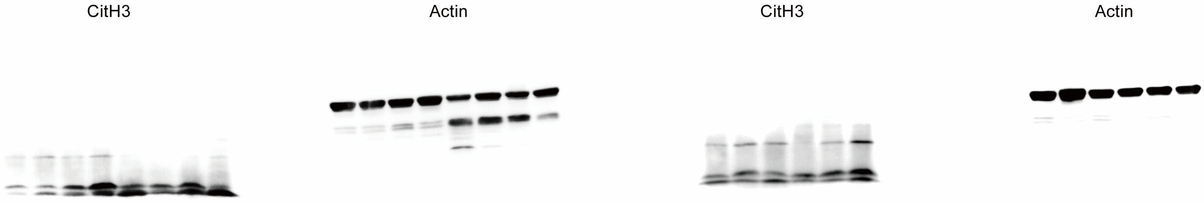

M Full unedited blot for Figure S4H

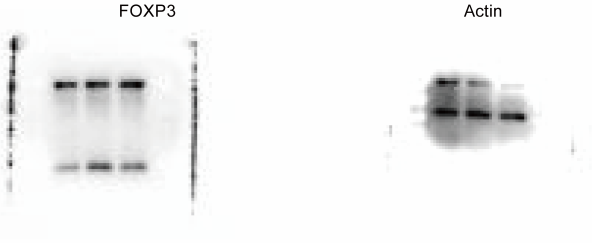

N Full unedited blot for Figure S5H

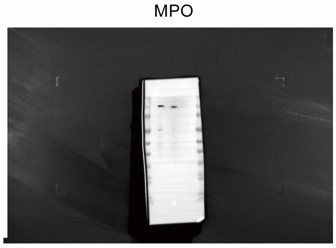

O Full unedited blot for Figure S5I

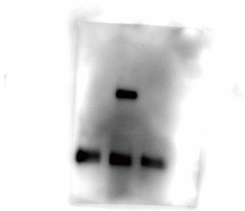

P Full unedited blot for Figure S7C

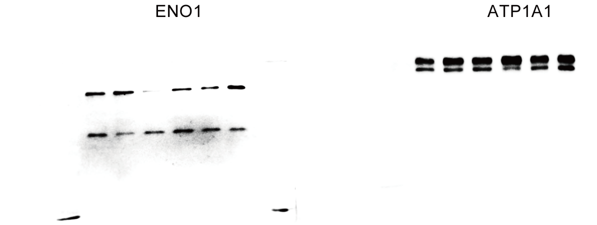

Q Full unedited blot for Figure S7H

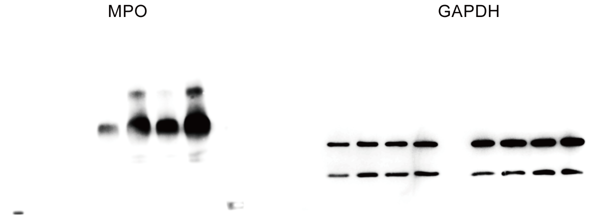

R Full unedited blot for Figure S7J

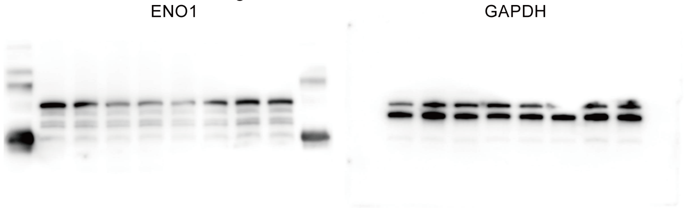

S Full unedited blot for Figure S8B

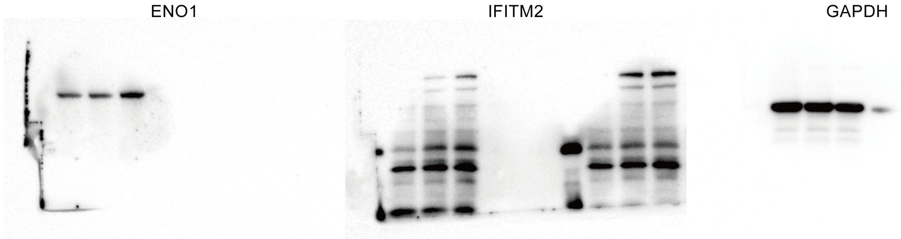

T Full unedited blot for Figure S8G

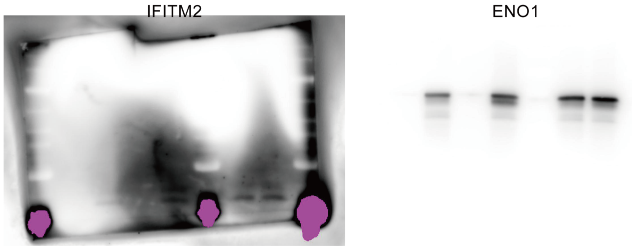

U Full unedited blot for Figure S8H

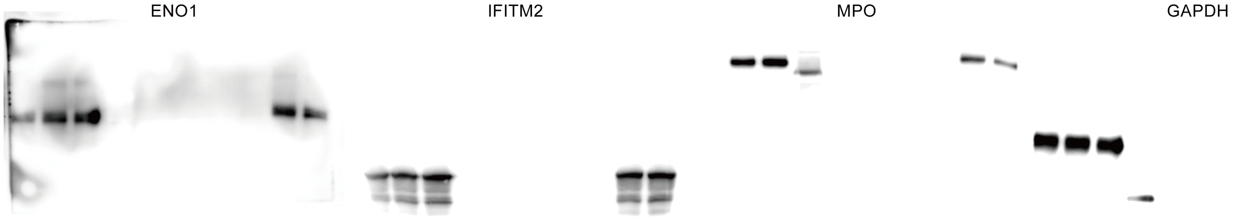

V Full unedited blot for Figure 9C

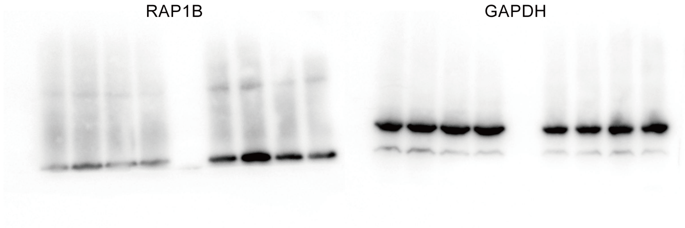

Supplement: Unedited blot and gel images [file jci-135-183541-s009.pdf]
